# Infinite hidden Markov models can dissect the complexities of learning

**DOI:** 10.1038/s41593-025-02130-x

**Published:** 2025-12-30

**Authors:** Sebastian A. Bruijns, International Brain Laboratory, Sebastian A. Bruijns, Petrina Y. P. Lau, Nathaniel J. Miska, Jean-Paul Noel, Alejandro Pan-Vazquez, Noam Roth, Karolina Z. Socha, Anne E. Urai, Kcénia Bougrova, Inês C. Laranjeira, Guido T. Meijer, Peter Dayan, Kcénia Bougrova, Inês C. Laranjeira, Petrina Y. P. Lau, Guido T. Meijer, Nathaniel J. Miska, Jean-Paul Noel, Alejandro Pan-Vazquez, Noam Roth, Karolina Z. Socha, Anne E. Urai, Peter Dayan

**Affiliations:** 1 Max Planck Institute for Biological Cybernetics, Tübingen, Germany; 2 University of Tübingen, Tübingen, Germany; 3 Champalimaud Foundation, Lisbon, Portugal; 4 University College London, London, United Kingdom; 5 The Chinese University of Hong Kong, Hong Kong, China; 6 Sainsbury Wellcome Centre, University College London, London, United Kingdom; 7 University of Minnesota, Minneapolis, MN USA; 8 Princeton University, Princeton, NJ USA; 9 University of Washington, Seattle, WA USA; 10 University of California, Los Angeles, Los Angeles, CA USA; 11 Leiden University, Leiden, the Netherlands

**Keywords:** Operant learning, Computational neuroscience

## Abstract

Learning the contingencies of a task is difficult. Individuals learn in an idiosyncratic manner, revising their approach multiple times as they explore and adapt. Quantitative characterization of these learning curves requires a model that can capture both new behaviors and slow changes in existing ones. Here we suggest a dynamic infinite hidden semi-Markov model, whose latent states are associated with specific components of behavior. This model can describe new behaviors by introducing new states and capture more modest adaptations through dynamics in existing states. We tested the model by fitting it to behavioral data of >100 mice learning a contrast-detection task. Although animals showed large interindividual differences while learning this task, most mice progressed through three stages of task understanding, new behavior often arose at session onset, and early response biases did not predict later ones. We thus provide a new tool for comprehensively capturing behavior during learning.

## Abstract (web summary)

Bruijns et al. present a modeling tool that enables the tracking of learning dynamics across subjects to reveal how behaviors emerge and adapt. Applying the tool to a decision-making task in mice uncovers similarities and differences across individuals.

## Main

Engaging with a new environment or task raises a multitude of problems—which sensory signals are pertinent to the task, and which are just noise? What actions are relevant to performance? How should observations inform actions? Particularly if the experimenter suddenly changes an aspect of the task (to manipulate or shape behavior), but also in stable environments, animals solve these problems through a mixture of apparent leaps in performance and slow accumulation of improvements^1–9^. This process of learning is marked by substantial variability across individuals, who progress at different speeds and over distinct intermediate stages^10^. Interindividual differences during learning are a known phenomenon^11^, although relatively little studied (although, for instance, see refs. ^12,13^). Even if the resulting behavior is highly similar across animals, variability during learning can make comparisons across groups in this period challenging^11^. This is because behavior during learning is a complex mixture of differently competent decision-making modes, prone to sudden shifts in performance (for better or worse), all of which occur on widely different timescales across individuals. More generally, the idiosyncrasies of the learning path may leave a trace in performance even after learning has finished and behavior has stabilized^14^.

Despite the richness of these dynamics, much of the work on the modeling of learning has ignored acquisition in its full breadth and generally considered only how animals adapt to ongoing changes in facets of tasks, such as reversing reward schedules. By this point in the task, the animals have learned the basics of the problem, and those that failed to learn it have been excluded. One of the reasons for this neglect of initial learning is that each animal provides only one sample of a learning curve, whereas for fully acquired behavior, every trial can typically be viewed as another sample from the learned behavior. This means that learning curve data are generally sparse, further aggravating the problem of large variability. Here we make use of the large-scale approach to data collection embodied by the International Brain Laboratory (IBL; ref. ^11^) to build a rigorous descriptive model of the multisession learning curves of more than 100 mice solving a perceptual decision-making task. While we used a large and varied dataset for development and testing, the final method does not require a large number of individuals and should therefore be broadly applicable to multisession learning data.

Previous work on task acquisition has sought to find the point in time at which an animal can be said to have ‘learned’ a task, often defined as reliably above chance performance^15^. Methods for solving this kind of change-point detection (for example, refs. ^16,17^) typically make a binary distinction between uninformed and learned behavior, rather than describing used strategies in detail, or finding possible intermediate stages. Other previous work addresses strategy inference more specifically and does consider learning^18^. This involves inference on a trial-by-trial basis over a set of simple, preselected strategies, decaying evidence exponentially over time to track the arrival and departure of various strategies.

We sought to accommodate the complexities of learning curves using a descriptive modeling framework that satisfies a number of desiderata. First, at any point along the curve, the model should capture an individual’s current repertoire of behaviors, characterizing its performance. Second, it needs to track this repertoire as behavior evolves, introducing new components (which we identify as behavioral ‘states’) when change is abrupt (for example, refs. ^9,19^), detecting the reuse of a past state if it re-emerges and allowing for slow, gradual shifts in a component, with the steady development of skilled performance (for example, ref. ^20^). Third, the collection of components should be potentially unbounded because we cannot know ahead of time how many distinct behaviors any individual might exhibit.

We therefore built a model that combines and extends two recent approaches. One is from ref. ^21^ (additional related work in ref. ^22^), which describes decision-making performance after learning with a hidden Markov model (HMM). Each latent state of the model captures a single component of behavior as a map from task-relevant variables to a distribution over choices, via logistic regression. In the case of perceptual decision-making, this generalizes a psychometric function (PMF) to include other factors (for example, perseveration). The overall description of behavior is in terms of a mixture of different policies that can switch rapidly. However, the HMM approach assumes stationarity of behavior across time and is constrained to a fixed level of complexity by specifying the number of states a priori. This weakens its ability to characterize the dynamic and idiosyncratic progression through training. To address these issues, we adopted the HMM framework to capture abrupt changes, except that (1) the states come from a Bayesian nonparametric structure, allowing for a degree of behavioral complexity that is only constrained by an inbuilt Occam’s razor and enabling the introduction of new states for suddenly appearing new behaviors^23–27^; and (2) we used a semi-Markov model so that latent states can persist for nongeometrically distributed numbers of trials.

The second approach is that of ref. ^28^, which effectively considers only a single state but allows the logistic regression weights implemented by that state to be dynamic, tracking changes in behavior through appropriate updates to the weights. We used this so that the characteristics of our hidden states can evolve slowly, capturing the other prevalent form of acquisition of skilled performance.

Showcasing our model on behavioral data from the IBL task, described in ref. ^11^, we reveal that learning progresses over a small number of distinct stages that are present in almost all animals. These stages apparently correspond to the sequential acquisition of elements of the task—in our case, particularly associated with taking into account different aspects of the sensory environment inherent to the task. Although this pattern was shared across the mice, the duration and diversity of the stages differed greatly between individuals.

We first describe the IBL task and our way of characterizing the behavior mice exhibit; then we discuss the details of the model by studying a representative fit to one animal in detail; and finally, we conclude by summarizing the fits of our model to 134 subjects, highlighting similarities and differences across the population.

## Results

**Fig. 1:**
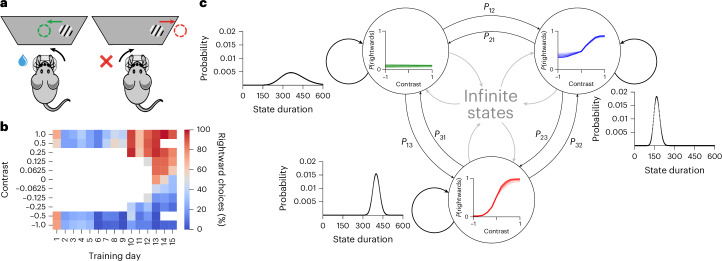
Behavior and modeling overview. **a**, Sensory decision-making paradigm. Mice indicated whether a contrast grating was on the left or right of a screen using a wheel. **b**, Representative behavior of mouse KS014 (also used later). This shows improvements in behavior and the concomitant extension of the contrast set. **c**, Visual representation of the main components of the model. Each state, represented by a circle, has an associated observation distribution, shown inside its circle. This is implemented via logistic regression, which considers the contrast on the current trial and a weighted history of previous choices (the latter is not shown here). The weights underlying these regressions can change from session to session, resulting in shifts of the PMFs they represent; we depict this evolution here with varying shades of color. States are connected to other existing states via transition probabilities *P*. In addition to that, states also have the option to transition into a new state, to describe a type of behavior that is not well captured by any of the existing states. Finally, staying in the same state for more than one trial is not modeled via a self-transition probability; instead, each state has its own duration distribution. Panel **a** reproduced from ref. ^11^ under a Creative Commons licence CC BY 4.0.

We analyzed the choices of 134 mice learning a perceptual decision-making task, each of which underwent, on average, 24.4 sessions (total, >3,200) and ~14,800 trials (total, >1.9 million)^11^. In this task, head-fixed mice were shown a sinusoidal grating of a controlled contrast, which had equal probability of being on either the right or left side of a screen (Fig. 1a). They then had to center it (within 60 s) by turning a steering wheel in the appropriate direction. Successful trials led to water reward, whereas unsuccessful trials resulted in a noise burst and a 1-s timeout. Trials were self-paced, with mice signaling their readiness by keeping the wheel still for a period.

Mice learned the task according to a shaping protocol that gradually introduced more difficult stimuli and actively removed action biases (Fig. 1b). Accordingly, shaping began with strong contrasts—100% and 50%. At the initial stage, there was no perceptual difficulty; the animals only had to learn the basic contingencies of the task. Once they had reached sufficient performance on these contrasts (≥80% correct for each contrast type on the last 50 trials), 25% contrasts were introduced. After performance was also good on this extended set (same criterion), the remaining contrasts were introduced in a staggered manner—12.5%, 6.125% and 0%, whereas the 50% contrast was dropped. For the 0% contrast, one side was randomly rewarded (50% probability per trial). A debiasing protocol increased the probability of repeating the stimulus that was just shown when the mouse made a mistake on an easy (100% or 50%) contrast. This deterred perseverative or biased strategies, but could lead to reward rates <50%.

To characterize the course of learning across trials, we developed a flexible model that segments an animal’s behavior into discrete states that last for variable numbers of trials within a session and can recur across multiple sessions. As this is a descriptive model, we equate a behavior with its corresponding state and, generally, will not distinguish between the two in the text. We first describe how a single state generates choice probabilities on a trial for which it was responsible (Fig. 1c, within circles) and then how we treat multiple states (Fig. 1c, arrows).

As in previous work^21,28^, we formalize the response probabilities for the binary choices of mice through logistic regression (omitting the rare trials in which the animal timed out by not responding within 60 s). Trial *t* of session *n* is described by features **f**_*n*,*t*_ comprising (1) the stimulus, that is, the contrast on the left and right of the screen, separated to allow for different sensitivities to leftwards and rightwards stimuli, as mice were frequently differently sensitive to the screen sides in this task; (2) task history, in the shape of an exponentially decaying average over the last actions—interestingly, mice only used a perseverative bias, but did not use reward information to implement a win-stay lose-shift strategy (as was also observed in refs. ^29,30^ and, in the same task at a later stage, in ref. ^31^); and (3) a bias term to allow for side preferences regardless of other features. Labeling the state that is active on this trial as *x*_*n*,*t*_, the response *y*_*n*,*t*_ ∈ {L, R} (for left and right) is modeled by the distribution

1 $$P(\,{y}_{n,t}={{R}})={\rm{sig}}\left(\,{{\boldsymbol{{f}}}}_{n,t}\times {{\boldsymbol{{w}}}}_{{x}_{n,t},n}\right),$$

where the weights of the states **w**_*x*,*n*_, ∀*x* are also indexed by session *n*, as they can drift across sessions. Here sig(⋅) is the standard logistic sigmoid function.

The model generalizes a standard HMM in the following three ways that make it especially suited to describe the phases of learning: (1) it is nonparametric about the number of states; that is, the number of states describing the behavior of each individual is separately determined, accommodating interindividual differences. This characteristic also allows the model to capture sudden changes in behavior, as it is able to introduce a new state when behavior changes notably (we call this the ‘fast process’; ‘Infinite hidden semi-Markov model’ section). (2) States are dynamic over sessions, allowing the behavior implied by a state to change gradually across session boundaries^28^ (the ‘slow process’; ‘Dynamic logistic regression prior and sampling’ section). (3) While for HMMs the numbers of trials for which a single state remains active always follow a geometric distribution, we adopt a semi-Markovian approach, allowing for more general distributions. Taking all these additions together, we end up with a dynamic infinite input–output hidden semi-Markov model (diHMM).

The transition matrix over a flexible number of states and the evolution of the psychometric weights are defined by priors, and the Bernoulli observation model provides a likelihood for each trial, allowing for approximate Bayesian inference (Methods). We performed this using a Markov chain Monte Carlo (MCMC) algorithm, namely Gibbs sampling. For a single animal, the entire response and feature data across all training sessions were fitted together. Individuals were fitted separately, meaning a large number of subjects is not necessary for the application of our model. Integrating across a number of Gibbs samples from multiple Markov chains led to a set of behavioral states defined by their session-varying weights **w**_*x*,*n*_ and duration distributions, as well as a hard assignment of every trial onto one of these states. While all other relevant random variables are specified hierarchically or ruled by vague priors, the variance for slow changes within states is set, as inference over this variable proved problematic; we revisit this parameter in the discussion.

### Single animal fit

**Fig. 2:**
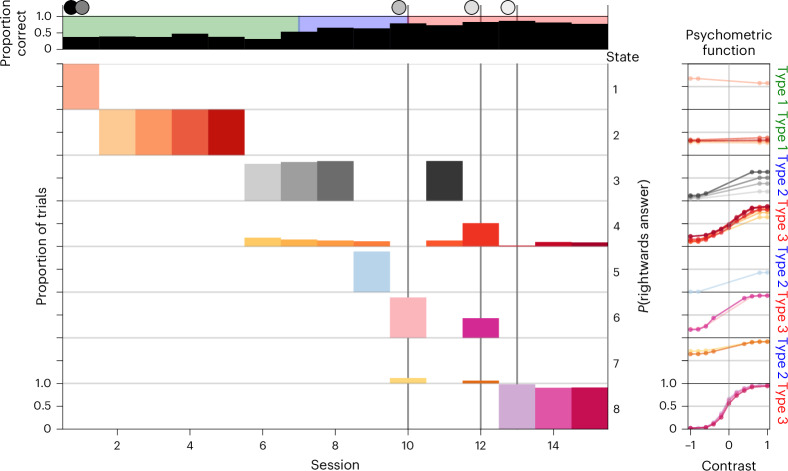
diHMM fit to mouse KS014. The topmost row shows the overall performance during the session, as proportion correct, and the current stage of learning as the background color (we elaborate on learning stages later in the text). Vertical lines with shaded circles at the top indicate the sessions during which new contrasts were introduced. The remaining rows show the prevalent behavioral states (label to the right) ordered by appearance, indicating which proportion of trials they explained during each session. To the far right of every state, we show its PMFs across time, ignoring the contribution from the history of previous choices. The saturation of the colors of the states indicates successive appearance and matches the PMF plots.

We visually summarize the model fit for mouse KS014 at the resolution of entire sessions in Fig. 2. This animal exemplified many of the interesting properties found across the population. The inferred model contains eight states, but these states were generally active for only a small number of sessions before being replaced by others. We number them in order of appearance. In a typical session, the majority of trials were explained by a single one; at most, a few were active. Later states generally represented more adept behavior, although not exclusively. The mouse started with state 1, which exhibited a flat PMF (far right of the plot), indicating that the animal did not take into account the side of the sensory input. This state was promptly replaced by state 2 in the next session, which also had a flat PMF, although shifted. This change in bias was strong enough to warrant a new state (rather than the slow process of changing the existing state), but there was no evidence that the animal advanced in its understanding of the underlying task.

State 2 lasted four sessions, indicating that behavior remained relatively consistent during this time. It was then predominantly replaced by state 3, which started with a mostly flat and strongly biased PMF (leading to a lower reward rate due to the bias correction) but improved considerably over the next few sessions, as can be seen in the evolving PMF (with darker colors showing later sessions). It seemingly considered only sensory information from the left side when making its choice, becoming increasingly random when that side was uninformative. The random behavior was doubly beneficial, as the animal would sometimes have been correct and also got foiled less by the bias-correction protocol. State 3 was accompanied by state 4, which described the behavior at the ends of the next few sessions (and later also at the ends of sessions 14 and 15). Puzzlingly, this state had a good PMF on both sides and a higher reward rate than state 3, but although this better state was available, the animal seemed incapable or unwilling to use it for the majority of a session.

The last major step in learning appeared abruptly as state 6, with good performance on both sides (albeit differently from state 4). Along with state 6, we observed the introduction of state 7, which captured a strong but transient decline in behavioral quality. Finally, state 8 represented another notable change in behavior, as performance on 100% contrasts increased abruptly enough to warrant a new state, allowing the mouse to conclude this part of training.

Various aspects of our model cannot be reproduced by existing treatments. The Psytrack model of the study discussed in ref. ^28^ can fit incremental changes in behavioral characteristics; however, because it lacks a concept of state, it does not natively support the identification of recurring behavioral patterns. We find that many states occur, then disappear, before reoccurring in a later session, such as states 3 and 6 in the animal shown in Fig. 2. This re-emergence of previously used strategies is an important feature of learning. Similarly, the static generalized linear model (GLM)–HMM described in ref. ^21^, which is aimed at asymptotic behavior, does not determine the number of states automatically. This implies that model selection is required for each individual animal, which the relatively small number of data points can make challenging. Furthermore, in the GLM–HMM, states cannot adapt their PMFs, which is a second important feature of learning. Without this, the GLM–HMM would tend to split states when behavior changes gradually but sufficiently as to elude a single set of weights.

**Fig. 3:**
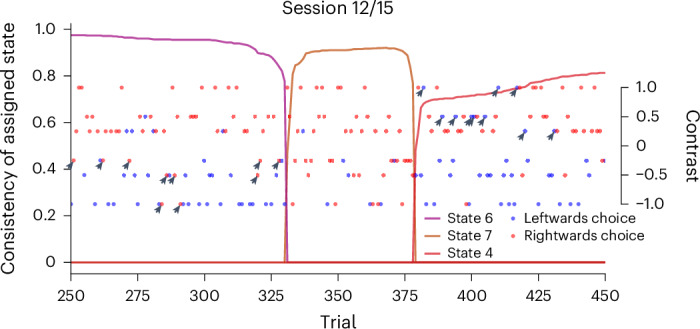
Excerpt of state assignments in session 12 from mouse KS014, also shown in Fig. 2. The left *y* axis serves as a scale for how connected a trial is to the other trials of that state (see Methods for details). The right *y* axis shows the contrast. The dot color indicates the animal’s response. One can see how the drastic and sudden change in the response patterns, rightwards (red) answers for leftwards (negative) contrasts, from trial ~330 to ~380 was detected by the model with a transition to state 7. The PMFs of states 4 and 6 looked similar but did, in fact, represent significantly higher error rates on the right and left sides, respectively. These mistakes are highlighted with arrows.

Our model also provides a fine-grained view of the use of behavioral states within a session. Although the diHMM provides a full posterior over the states for each trial, this is not directly useful due to the technicalities of the sampling procedure. We therefore processed the sample chains to estimate how much a trial belonged to a state (Methods). We show an excerpt of this, for session 12 of mouse KS014, in Fig. 3. This shows two clear transitions between states. The reasons for the animal to have made such a transition are probably multifaceted and may have been both internal (for example, insights or motivational fluctuations^32^) and external (for example, a number of low contrast, perchance unrewarded trials demotivating the animal). We do not model these reasons and, instead, only describe observed changes.

The within-session fit shows that the model can detect temporary yet strong deviations in behavior. State 7 only explained a couple of dozen trials in two sessions, but represented extremely biased behavior (comparable, but flipped relative to the earlier state 2, albeit lasting for many fewer trials). We speculate that state 7 arose from a form of inattention because the animal had previously shown itself capable of performing appropriately. This change in behavior is directly evident in the response patterns of the animal.

We can also capture subtler differences in behavior. The model used different states to explain behavior before and after state 7, although performance appeared equally good. However, the model identified different error rates on easy contrasts for the two states, and this can be found in the choices—state 6 was associated with more incorrect responses to contrasts on the left side, whereas in state 4, performance on leftwards contrasts was good, but there were frequent lapses on rightwards contrasts (comparing two logistic regression models—one using contrast and state 4 (assigned to *n* = 391 trials) or 6 information (assigned to *n* = 331 trials) to predict responses, and the other a nested model that only uses contrast (for the total of *n* = 722 trials)—the state-split model is significantly better, as determined by a likelihood-ratio test with *P* < 0.0006 (*D* = 14.96, *df* = 2), with an effect size, as measured by the McFadden pseudo-*R*^2^, of 0.025; Fig. 3, responses marked by arrows).

### Fits across the population

**Fig. 4:**
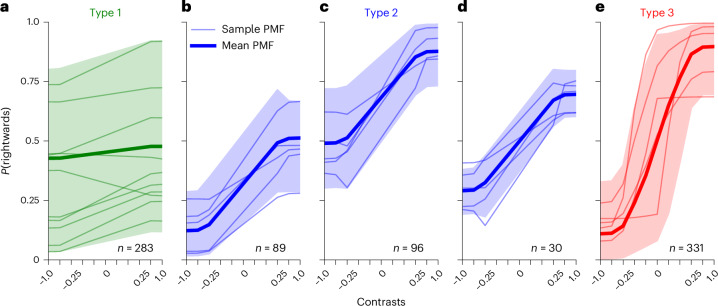
Summary of the PMFs associated with the different types. **a**–**e**, The first PMF of each state in each animal (representing response characteristics after a notable discontinuity in behavior) was collected. Each subplot shows a specific type—type 1 in green (**a**); type 2 in blue, further split by whether the PMF is left-biased (**b**), right-biased (**c**) or symmetric (**d**); and type 3 in red (**e**). The thick lines indicate the overall mean over PMFs of the type, which shows representative behavior of that type. The shaded regions show the range in which 95% of the PMFs fell (computed separately for each contrast level). The thin lines show samples of individual PMFs of these types. ‘*n*’ indicates how many PMFs of each type were present across the entire population.

The threefold progression we observed throughout learning in Fig. 2—from flat PMFs, to ‘one-sided’ behavior, to generally good performance—is typical for the population of mice we fitted. To define this more objectively, we clustered the states into these three types based on their reward rate on easy trials (see ‘Psychometric type classification’ section for details). The boundary between types 1 and 2 is at a 60% reward rate, and the boundary between types 2 and 3 is at a 78% reward rate. We show an overview and examples of the different types in Fig. 4.

In addition to the state types, we define the stage at which an animal is on any given session as the highest type it has so far used for the majority of trials of any session up to this point. For instance, if up to session *n* − 1, an animal only used type 1 states or type 2 states for fewer than 50% of trials, then it would be in stage 1 for those sessions. If, on session *n*, it then used type 2 states for more than 50% of trials, it would switch to stage 2 on that session. Because the state types delineate different aspects of task understanding, the stages allow us to determine how many sessions the animals stayed at a certain level of understanding. While the progression through state types was not monotonic (for example, session 11 of Fig. 2), the stage classification is, by definition, monotonically increasing.

Stage 1 consisted of states with flat PMFs of various biases, generally ignoring the contrast location. Stage 2 almost always involved asymmetric states, responding well to one side of the screen, but close to uniform guessing for the other (PMFs assigned to Fig. 4b,c account for 86% of those in stage 2; see ‘Psychometric type classification’ section for details). Only rarely were intermediate PMFs nearly equally good on both sides (the 14% in Fig. 4d). These rare cases did behave like the other type 2 states in terms of their time of appearance during training as well, rather than type 3. Finally, in stage 3, the animals started apparently paying attention to both sides. Generally, it took some further refinement of initial type 3 states, through the reduction of errors on easy trials on either side, to master this stage of training and progress to the next phase of shaping.

**Fig. 5:**
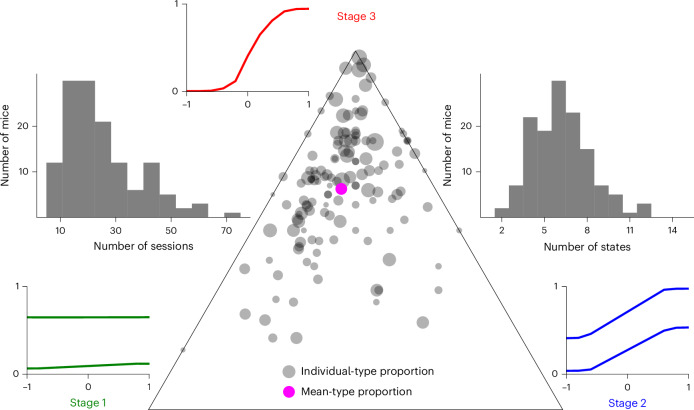
Proportions of sessions it took each mouse to reach the next major step in training, as defined by the three stages. Each individual is represented as a circle on the simplex (the larger the proportion of sessions within a specific stage, the closer the dot for that animal is to that corner of the simplex). Simplex corners are identified by example PMFs of the stage type. The marker area indicates the total number of sessions (min, 5; max, 75). The magenta circle marks the average proportion, and its size indicates the mean number of sessions (which was 24.4). See Supplementary Fig. 1 for a linear version of this plot. The histogram on the right shows the distribution over the number of states used by the model per mouse. The histogram on the left shows the distribution over the number of training sessions.

The three stages segment the learning process. We can analyze the proportion of training time the animals spent in the different stages by showing these proportions on a simplex (Fig. 5 and Supplementary Fig. 1, linear representation). The large majority of animals spent some time in each of the stages (that is, only a few mice are assigned to the edges of the simplex). Most animals spent the longest time in stage 3—going from moderately competent performance to passing the stringent training criteria. No fundamental change in understanding was necessary for this, unlike the changes from stage 1 to 2 or 2 to 3 (where the animal had presumably to learn to pay attention to the Gabor patch on one or both sides of the screen). However, reaching the required accuracy seemed difficult, even once the principles of the task were understood (possibly due to the small increase in reward rate afforded by the extra accuracy). Some of the longest trajectories (the largest circles) were associated with especially many sessions in stage 3, but overall the average fractional occupation was remarkably consistent across training lengths (the mean relative occupancy for stage 1, 2 and 3, respectively, were (0.24, 0.17, 0.59) and (0.21, 0.14, 0.65) for the shorter and longer halves of a median split on the total number of training sessions). Stage 2 consistently lasted for the fewest sessions, implying that the mice managed to pay attention to both sides not too long after starting to pay attention to one side.

**Fig. 6:**
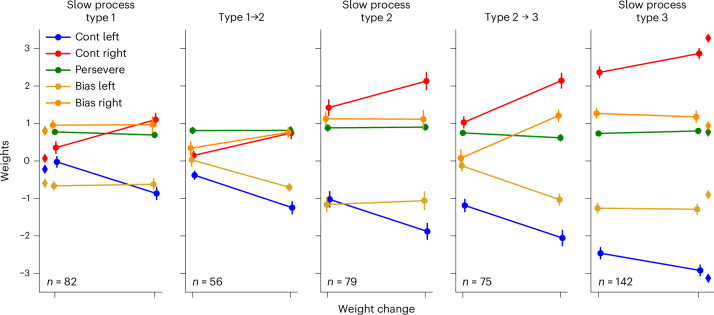
Evolution of the weights of states on average, through slow and sudden changes. Error bars indicate ±1 s.e.m. (lines are slightly offset along the *x* axis for visibility). Subplots titled by a type represent the weight changes from the first appearance of a state of this type to its last, so only showing state-internal slow changes (and only including states that were present between 5 and 15 sessions, as extremes would skew these averages). Subplots with a title indicating a transition from one type to another show how much each weight of the new state differed from the weights of the closest previously existing state and are based exclusively on the states that first brought the mouse into a new stage. That is, for ‘type 1→2’, we only took into account the first type 2 state exhibited by the mouse and only when that state was type 2 from its inception. For instance, for mouse KS014, this was state 4, which started as type 2 before using the slow process to become type 3. Colored diamond markers on the leftmost and rightmost plots indicate the average value of the weights of the very first state of each mouse and of the dominant state on the last session, respectively. To prevent biases from canceling out across the population, we split the bias weights into the following two groups: starting out below 0 (bias left) or starting out above 0 (bias right). While contrast sensitivities increased both through fast and slow changes, it is noticeable that biases stayed almost constant throughout the lifetime of a state on average, but changed more noticeably through sudden transitions.

Connected to this is the question of how slow and fast changes characterize behavior. We analyzed gradual changes within a state by comparing its PMF weights on its first and last appearances. We analyzed new state introductions by comparing their PMF weights to those of the closest previous state, as determined by the Wasserstein metric on their resulting PMFs (ignoring the perseverative weight). To highlight the changes, we focused on states that brought the animal into a new stage. These weight evolutions, split by type, are shown in Fig. 6. As the main driver of performance, contrast sensitivities reliably increased both over the lifetime of a state and when new states were introduced. Surprisingly, however, both the bias and perseverative weights were stable within a state. This was markedly different for the fast process—the changes through this were significantly larger (Supplementary Figs. 12 and 13; one-sided Mann–Whitney *U* test on absolute weight changes, two biases, two fast change points, three slow change processes; the fast process had significantly larger changes for all 12 comparisons at a 0.05 significance level, after applying the Benjamini–Hochberg procedure to control the false discovery rate, with effect sizes ranging from 0.43 to 0.94, quantified as standardized mean difference, using the fast change s.d.). We also see that the perseveration weight had a small but consistent role throughout learning (although its relative influence waned as the sensitivities grew).

**Fig. 7:**
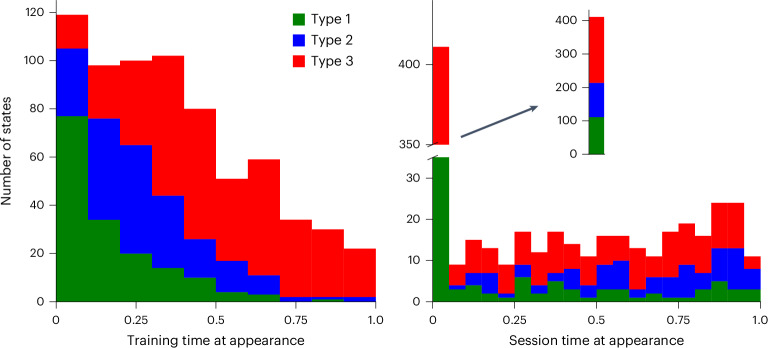
Histograms of all state introductions. The first state of every animal (which necessarily occurred on the first trial of the first session) was excluded. State introductions are shown across all of the training sessions (left) and within sessions (right). We color by state type (green, blue and red for types 1, 2 and 3, respectively) and normalize the entire length of training of an animal, as well as all individual sessions, onto the range between 0 and 1 for comparison purposes. The inset on the right plot shows the bar of the first time bin uninterrupted.

The introduction of new states signifies notable changes in behavior, so by studying the patterns of their occurrences, we gain insight into when behavior was volatile or when substantial progress was made. The histograms in Fig. 7 show when new states first appeared across normalized training and session times. In later sessions, gradually fewer states were introduced, indicating that behavior saw fewer drastic changes as training progressed. We noted earlier that animals spent most of their time in stage 3, that is, perfecting their behavior, and we can now conclude that gradual improvements had an important role in this, more so than sudden marked changes. The pattern of introductions within sessions is even more striking—the majority of states were introduced at the very start of a session. This resonates with previous findings about change points in behavior occurring at session boundaries^16^.

#### Interindividual differences and variability

So far, we have highlighted general patterns during learning, but perhaps even more salient than these similarities was the wide-ranging variability across animals. Such differences are already visible in many of the plots above. Biases in type 1 states spanned the entire range of possible response patterns. Similarly, type 2 PMFs appeared to be randomly biased toward one side or the other, or, rarely, symmetric. We were particularly surprised to find no regularity between type 1 and type 2 biases. Of the 56 mice in which type 2 onset occurred suddenly, 31 had expressed the same direction of bias (average choice from the PMF being more than 5% away from 50%) as the new type 2 state in any previous type 1 state, whereas 25 had not (two-sided binomial test for whether the proportion of previously expressed biases differs from 0.5 gives *P* = 0.504). Thus, we were unable to predict future biases of the animal from its stage 1 biases.

**Extended Data Fig. 1:**
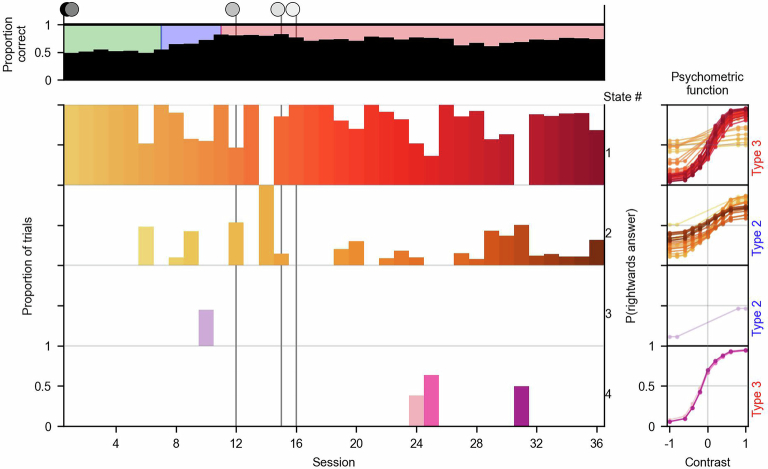
Model fit to a mouse with a larger number of sessions. Using the plotting conventions of Fig. 2, this depicts the states and corresponding psychometric functions identified in a mouse that required 36 sessions to learn. This illustrates the counterintuitive phenomenon that long training trajectories were sometimes fitted by the model using a small number of states. Some of these states, particularly state 1 in this example, underwent substantial changes through the slow process, spanning the range of uninformed to proficient behavior (note that the type labels to the right of the PMFs are determined by the highest type reached by a state; thus, state 1 is labeled as type 3 even though it began as type 1).

The number of sessions mice required to learn varied greatly, spanning an order of magnitude. Surprisingly, many animals with a large number of sessions were fitted by a small number of states, which changed considerably via the slow process, as exemplified in Extended Data Fig. 1 (notably, our recovery analyses indicate that the model can cope effectively with long training trajectories, as described in Methods). We revisit this issue in the ‘Discussion’.

The number of sessions spent in the different stages was similarly highly variable. To gain insight into the factors underlying the learning steps between the stages, we analyzed the correlations between the number of sessions spent in them. The simplex plot does not strongly indicate any patterns. We quantify this as follows: duration of stage 1 to stage 2—Pearson’s *r* = 0.21, *P* = 0.015; stage 1 to stage 3—Pearson’s *r* = 0.04, *P* = 0.685; stage 2 to stage 3—Pearson’s *r* = 0.14, *P* = 0.095 (*n* = 134 mice). Notably, the main chunks of training time, stages 1 and 3, show no correlation whatsoever. A speedy understanding of the basic contingency of the task, therefore, did not tend to go along with the ability (or will) to perfect this behavior quickly, suggesting that they required different competencies. The strongest correlation exists between stages 1 and 2, which makes sense insofar as they were both concerned with discovering how to make use of the stimulus information.

Beyond the training sessions analyzed here, the mice underwent a further phase (‘biased block training’, in which left or right stimuli dominated in blocks of 20–100 trials). Consistent with our other results, the length of this phase also turned out not to positively correlate with the total prebias training duration, nor with any of the stage durations. At most, there was a negative correlation between the overall bias training time and the stage 3 duration (see Supplementary Results for details).

## Discussion

We presented a highly flexible model that describes the stages of learning from the very first day an animal interacts with a task until it becomes an expert. Using it on the shaping sessions of the IBL decision-making task, we showcased a number of useful capabilities of this approach. It allowed us to distinguish fast, abrupt transitions in behavior, and slower, gradual ones. Learning on this task decomposed into the following three distinct stages, through which almost all animals went: initial, undifferentiated and often biased behavior; partial, one-sided understanding of task contingencies; and, finally, full understanding of the task. While these broad-stroke characteristics were consistent across mice, and indeed resonate with recent results from other tasks^33,34^, the details of behavior in these stages differed considerably across the population. Similarly, the way they progressed through these stages differed widely in both its duration and the composition of the sudden and gradual steps.

We found only a weak correlation between the time it took individual mice to progress through some of the behavioral stages, suggesting that they had to draw upon largely different skills to learn the requirements of the task. Similarly, animals expressed varying, largely uncorrelated, biases across the stages of learning. They might therefore have different sources—in stage 1, when the mice paid no attention to the stimulus, biases might be motoric; in stage 2, they could have been an expression of the side that individual mice happened to notice first as being informative; in stage 3, they might have stemmed from differences in sensory acuity. Beyond the initial training considered here, the duration of the subsequent biased block training of the animals did not exhibit positive correlations to the training phase durations (as elaborated in Supplementary Results). This again shows that learning was influenced by a large number of factors in our setting.

We originally expected that mice who took many sessions to train would be characterized by many states. However, although recovery analyses show that the model can cope effectively with long trajectories, this was not always the case. Instead, we often saw that few states took a long way via the slow process, from uninformed to proficient (Extended Data Fig. 1). It will be important to assess the underlying nature of these states and their progression by tracking neural data through the course of learning.

It is important to note that our model does not require as large a dataset as we used. Individuals were fitted by themselves, the model proved flexible enough to accommodate considerably different numbers of training sessions, and our cross-validation indicates that the fits are not critically sensitive to hyperparameter selection, the only part which made use of all subjects combined. Nevertheless, our modeling approach does have a number of limitations. First, the setting of the slow change variance parameter, which determined how much the behavior of a state could change from one session to the next, has a critical role in steering the trade-off between introducing a new state versus adapting an existing one. We optimized this parameter in terms of cross-validation performance for the entire population (Methods). However, the magnitude of slow changes may depend on the individual or vary across training time, and thus, a more differentiated treatment might be appropriate. Furthermore, slow changes may also occur within a session^28^, which could be incorporated into the model by adding additional time points at which weights can change. This might well be necessary to apply our method usefully to the sort of more rapid, continuous changes that occur within a single session. Another desirable extension would be to allow the duration distributions to change over sessions. As training progresses, an animal might, for instance, be able to use a highly performant state for longer. Similarly, a dynamic transition matrix and dynamic initial state distribution might better capture the evolution of state usage across training.

The model may be extended by making the states predict additional observations, as binary choice behavior may limit the power to distinguish between behavioral modes. One obvious possibility is the reaction times of the animal’s choices; in principle, this would only require adding a suitable distribution to produce times for each state (for example, from a drift diffusion decision-making process^35,36^). It would likely be necessary to make the distributions dynamic, as the reaction times improve with training. Other possibilities include pupil dilation or even body posture^37^.

Previous work using an HMM-based approach discovered demotivated states in behavior during the first 90 unbiased trials per session in the subsequent biased block training^21^. The prevalence of sizable blocks of trials during which the animal performs at a decreased level will, if left unaccounted for, lead to confounded estimates of model parameters and a flawed understanding of the animal’s current skill development stage, making it an integral component of a good behavioral model of this task. We also find such states, characterized by reduced sensitivity to the contrast feature of at least one side, and a strong bias in extreme cases, leading to higher-than-normal lapse rates on strong contrasts. However, these were not as pervasive as might have been expected from ref. ^21^. For us, a majority of sessions were dominated by a single state. The model sometimes acknowledged the dip in performance of the animals at the ends of sessions for tens of trials with a separate state (as shown in Fig. 2 on multiple sessions). We analyze aspects of these trials in ‘Posterior predictive checks’. However, frequently, we just see a decrease in the prevalence of all sufficiently represented states. The main source of behavioral variability in our data came from learning and other large jumps in psychometric space; therefore, the model used its capacity to capture these.

Besides task acquisition, our approach to capturing behavioral evolution, which has conceptual relations to those used in the animal conditioning^38,39^, structure learning^40^ and motor learning^8,41^ literatures, should be well suited to model other progressive changes, such as those occurring during ageing^42^. Furthermore, our framework can be flexibly adapted to other cases of long-run learning. For instance, it is possible to tune the model to capture minute changes within sessions rather than broad-stroke states across sessions, as here, by adjusting the propensity to infer new states for small changes in behavior. Equally, the modular resampling procedure of the model allows it to be adapted to different kinds of observations, for example, multinomial or Gaussian, by simply swapping out the inference mechanism of this component (although only some distributions are convenient for the gradual dynamics). We therefore hope that the tool we developed here will enable a wide range of researchers to study behavioral development in a systematic and revealing manner.

## Methods

In this section, the data source is described briefly, followed by a detailed explanation of the infinite hidden semi-Markov model. Inference for the logistic regression observation distributions is then covered, with a focus on the resampling steps. Together, these components make up the full diHMM. The aggregation of generated samples is then explained, addressing challenges such as label switching and multimodality to define clear states. The process for assigning states and their PMFs to the three types is described in ‘Psychometric type classification’ section. Finally, validation analyses are presented, including cross-validation for parameter and prior selection, model ablations, posterior predictive checks, and recovery of generative models.

### Ethics statement

All procedures and experiments were carried out in accordance with the local laws and approval was obtained from the following the relevant institutions: the Animal Welfare Ethical Review Body of University College London (P1DB285D8); the Institutional Animal Care and Use Committees of Cold Spring Harbor Laboratory (1411117; 19.5), Princeton University (1876-20) and University of California at Berkeley (AUP-2016-06-8860-1); the University Animal Welfare Committee of New York University (18-1502); the Portuguese Veterinary General Board (0421/0000/0000/2016-2019).

### Animals and behavioral data

The data we used were collected under the IBL protocol, as described in detail in ref. ^11^ and its accompanying materials. The study subjects were female and male C57BL6/J mice, aged 3–7 months, which were cohoused whenever possible. Mice were kept in a 12-h light/12-h dark cycle and fed a diet containing 5–6% fat and 18–20% protein. No statistical methods were used to predetermine our sample size, but the IBL represents a large-scale approach to data collection and offers an exceptionally large dataset of learning trajectories (covering more individuals than the studies on learning by, for example, refs. ^12,13^). There was no blinding of experimenters, as there were no experimental groups. The stimulus sides and strengths that animals were presented with were independently drawn for each session (although the debiasing protocol could affect these probabilities, and weaker contrasts were introduced in a performance-dependent manner). When using Pearson’s *r* to quantify correlation, the data distribution was assumed to be normal, but this was not formally tested.

### Infinite hidden semi-Markov model

We start by describing the diHMM, focusing on Bayesian inference over its random variables. Following ref. ^25^, we use Gibbs sampling, an MCMC algorithm, to realize an iterative resampling scheme over the model components, including the PMFs of the hidden states and the assignments of the individual trials onto those states. For this purpose, all distributions are paired up with conjugate priors in this section, to enable simple resampling steps. The posterior distribution is ultimately represented by a collection of samples, with every component being assigned an explicit value in each sample.

We first describe all the relevant random variables, using the iterator notation from Supplementary Table 1.

The technical backbone of an infinite HMM is a hierarchical Dirichlet process. At the top of the hierarchy of this process is the prototypical transition vector

2 $${{{\bf{\upbeta }}}} \sim {\rm{GEM}}(\gamma ),$$

where GEM (named after Griffiths, Engen and McCloskey) is a Dirichlet process without a base distribution, a pure stick-breaking process that samples a probability vector over infinitely many elements (which will be states in our case). The concentration parameter *γ* probabilistically determines the size of the individual sticks and, therefore, the number of practically relevant states, with higher *γ* encouraging more states. We put a vague Gamma prior on *γ*, making it, and thereby the propensity to introduce new states, part of the inference as well, with *γ* ~ Gamma(0.01, 0.01).

At the next level, we sample the transition vectors, a classical HMM component, **π**_*i*_, of the individual states *i*. These are tied together via **β**, which is used as the base distribution for a second Dirichlet process

3 $${\pmb{\uppi}}_{i} \sim {\rm{DP}}(\alpha ,{\pmb{\upbeta}}),\qquad i=1,2,\ldots ,L,$$

4 $${\pmb{\uppi}}_{{\bf{0}}} \sim {\rm{GEM}}(3).$$

*α* is another concentration parameter and determines how closely the **π**_*i*_ are related to **β**. Sampling the individual state transition vectors from this common source formalizes an overall kind of state popularity. The higher *α*, the more like **β** is **π**_*i*_, ∀*i*, and so the more the bias in the frequency of state ${i}^{{\prime} }$ in the particular sample **β** will be reflected in the transitions from *i* to ${i}^{{\prime} }$, and so the more popular ${i}^{{\prime} }$ will be overall. We put another vague Gamma prior on it, *α* ~ Gamma(0.01, 0.01). The initial state distribution **π**_0_ is drawn entirely separately, with a concentration parameter of 3 as a trade-off between allowing new states but not encouraging the invention of new states at the start of sessions.

For our inference scheme, we make use of the weak-limit approximation, which puts an upper limit *L* = 15 on the number of states, rather than using the full infinite process. This simplifies the resampling scheme, while still behaving similarly to an infinite HMM if *L* is sufficiently large. Across the entire population, there were only three mice with 12 states, after applying our hierarchical state clustering procedure (‘Aggregation and interpretation of chains’ section); all other mice used fewer states. Furthermore, the minimum fraction of trials captured in states (as described further below) is 99.38% (mean = 99.97%), justifying the choice of *L* = 15 (although a higher limit would possibly allow us to capture motivational fluctuations better). In particular, we still perform inference over the realized state complexity. In the weak-limit framework, equations (2)–(4) turn into *L*-dimensional Dirichlet distributions

5 $${{\bf{\upbeta }}} \sim {\rm{Dir}}(\gamma /L,\ldots \gamma /L),$$

6 $${{{\pmb{\uppi }}}}_{{{i}}} \sim {\rm{Dir}}(\alpha {{{\bf{\upbeta }}}}_{1},\ldots ,\alpha {{{\bf{\upbeta }}}}_{L}),\qquad i=1,2,\ldots ,L,$$

7 $${{{\pmb{\uppi }}}}_{{\rm{0}}} \sim {\rm{Dir}}(3/L,\ldots ,3/L).$$

The transition structure within a session is given by

8 $${z}_{n,1} \sim {{{\pmb{\uppi }}}}_{\rm{0}},$$

9 $${z}_{n,s} \sim {{{\pmb{\uppi }}}}_{{z}_{n,s-1}},$$

where *z*_*n*,*s*_ ∈ {1…*L*} is an indicator for the *s*th state within a session *n* (which does not align with the trial number), and **π**_0_ is the initial state distribution.

Given the transition vectors, the workings of the hidden semi-Markov model are fairly standard, except that the duration distributions are specified explicitly rather than being drawn from a geometric distribution (as in a regular HMM). We therefore prohibit self-transitions, which makes a data-augmentation scheme for resampling necessary, as described in ref. ^25^. Nevertheless, as in a standard HMM, durations are statistically independent of the target state of transitions. Durations are drawn from a negative-binomial distribution, with state-specific random variables, coming from their own priors

10 $${r}_{i} \sim U(5,6,7,\ldots ,704),\qquad i=1,2,\ldots ,L,$$

11 $${p}_{i} \sim {{\rm{Beta}}}(1,1),$$

12 $${d}_{n,s} \sim {\rm{NB}}({r}_{{z}_{n,s}},{p}_{{z}_{n,s}}).$$

Note the difference between state names *i*, which hold for the entire model, and the session-specific state counters *s*, which can be used to find the current state name via the indicator *z*_*n*,*s*_. We chose a uniform prior over a large range of numbers for the possible values of *r*, to enable long durations, but excluded small values for *r* (in particular, *r* = 1 would give the geometric distribution). Small values of *r* encourage transitions after a very small number of trials, which would capture the statistics of the presentation of left and right stimuli by the experimenter rather than the longer-lasting states that we sought. Using cross-validation, we ensured that enabling larger values of *r* did not benefit the fits.

States stay active and generate observations for as long as the drawn duration indicates

13 $${t}_{n}(s)=\mathop{\sum }\limits_{k=1}^{k < s}{d}_{n,k},$$

14 $${x}_{n,{t}_{n}(s)+1:{t}_{n}(s)+{d}_{s}}={z}_{n,s}$$

15 $$P(\,{y}_{n,t}={{R}})={\rm{sig}}\left({{\mathbf{{f}}}}_{n,t}\times {{\mathbf{{w}}}}_{{x}_{n,t},n}\right),$$

where we defined *t*_*n*_(*s*) to return the trial on which the *s*th state of a session *n* starts, which allows for the definition of *x*_*n*,*t*_, the state on any given trial *t*. We denote the logistic sigmoid function as sig. This takes the dot product between the state weights **w**_*s*,*n*_ (which we discuss in the next section) and the input features of the current trial **f**_*n*,*t*_ and produces the probability over the observation *y*_*n*,*t*_. The binary response variable *y* has 0 representing a leftward, and 1 a rightward choice. See also Supplementary Fig. 2 for a visual summary of these variables.

We summarize this collection of variables as

$$\Theta =\left\{\gamma ,\alpha ,{\beta} ,{\pmb{\uppi}}_{0},{\{{\pmb{\uppi}}_{i},{r}_{i},{p}_{i},{\{{{\mathbf{{w}}}}_{i,n}\}}_{n = 1}^{N}\}}_{i = 1}^{L},{\{{\{{x}_{n,t}\}}_{n = 1}^{{N}_{t}}\}}_{t = 1}^{T}\right\},$$

where *N* is the total number of sessions. The connections between these variables are visualized in the form of a graphical model in Supplementary Fig. 3. The result of inference is a set of samples ${\{{\Theta }_{j}\}}_{j = 1}^{J}$. Each sample is a full instantiation of the listed random variables, which we can treat as a posterior representation. Gibbs sampling works by iteratively sampling each variable from its distribution, given all other variables in the model. After updating all variables, the result is one new sample within the MCMC chain. Details on how to resample the individual components can be found in ref. ^25^.

### Dynamic logistic regression prior and sampling

Gibbs sampling resamples each random variable conditioned on all others. Thus, inference over the observation distributions of the states is separate from almost all the rest of the model, only using the information as to which trial is currently assigned to which state. We drop the explicit state dependence *i* in **w**_*i*,*t*_ for this section, but it is important to keep in mind that this sampling scheme is applied to every state individually, with each state *s* being influenced only by trials for which *x*_*n*,*t*_ = *s* in the current sample. We implement slow changes in the characteristics of the states by putting a Gaussian random walk prior on the weights **w**_*n*_, allowing for modest change across session boundaries, parameterized by the variance *σ*. We choose a diffuse initial distribution for the weights and use cross-validation to select the intersession variance *σ* = 0.04 (we performed cross-validation on a range of small values, to limit the state adaptation process to small changes)

16 $${{\mathbf{{w}}}}_{1} \sim \mathcal{N}(0,8\ I),$$

17 $${{\mathbf{{w}}}}_{n+1} \sim \mathcal{N}({{\mathbf{{w}}}}_{n},\sigma \ I),$$

where *I* denotes the identity matrix. If a state has no trial assigned to it in a particular session, its weights are held fixed during the next transition, preventing states from morphing radically during a prolonged absence.

Inference for the logistic regression weights is performed using Pólya-Gamma data-augmentation, which allows for efficient inference in settings with binomial likelihoods^43,44^, because it is not possible to choose a conjugate prior. We review the relevant computations here; for a full treatment, we refer to ref. ^45^. In the first step of the resampling scheme, we sample pseudo-observations. This uses a Pólya-Gamma distribution PG, by first sampling *ω*_*n*_ ~ PG(*b*_*n*_, *ψ*_*n*_), where *ψ*_*n*_ = **f**_*n*_ × **w**_*n*_ is the dot product of features and weights, and *b*_*n*_ is the total number of times this exact instantiation of features was observed in session *n*. However, the same state is associated with more than just one specific instantiation of features (that is, including contrasts of different strengths and sides and different response histories). To handle this, we treat a single session as multiple different time points, but prevent weight changes between time points that belong to the same session. In this way, the observations from different features within the same session are effectively aggregated. To complete the pseudo-observation generation, we need *κ*_*n*_ = *a*_*n*_ − *b*_*n*_/2, where *a*_*n*_ is the number of rightward answers observed for the current *ψ*_*n*_ under consideration. Now *z*_*n*_ = *κ*_*n*_/*ω*_*n*_ can be treated as if they were drawn from $\mathcal{N}({{{\psi }}}_{n},1/{\omega }_{n})$.

This data-augmentation serves the purpose of having the **w**_*n*_ emit observations with Gaussian noise (after combination with the features **f**_*n*_ into *ψ*_*n*_). Because the prior on **w** is a Gaussian random walk, this places inference in the well-studied realm of Kalman filtering. To resample the **w**_*n*_, we use the forward filter backward sample algorithm^46,47^, which filters forward through all the observations using a Kalman filter, then samples the sequence of **w**_*n*_ backwards through time. A single resampling step, therefore, consists of first drawing the Pólya-Gamma variables to create pseudo-observations, then using them to sample the **w**_*n*_ using the forward filter backward sample algorithm.

We consider four features for the logistic regression—the contrast on the left side, the contrast on the right side, an exponentially weighted history over all previous choices and a bias. Separating the features for left and right contrast allows the sensitivities to the two sides to be different. Because the notional contrast values do not match the psychophysical difficulty of the contrasts (100% and 50% are both virtually equally easy to perceive, not a factor of 2 apart), we apply a transformation to have a better alignment. For this, we follow ref. ^28^ and use a tanh transformation, mapping the actual contrast *c* onto the input $\tilde{c}$ for our logistic regression through $\tilde{c}=\tanh (pc)/\tanh (p)$, where we follow their recommendation and set *P* = 5, which scales the steepness of the transformation. This maps the contrasts (1, 0.5, 0.25, 0.125, 0.0625, 0) onto (1, 0.987, 0.848, 0.555, 0.302, 0).

The regressor for previous answers, enabling perseveration as a strategy, proved to be beneficial for cross-validated performance. It is associated with the famous law of exercise^48,49^ and has also been found to be exhibited by the mice in the asymptotic regime that arises after the sessions that we are presently analyzing^31^. The same analyses showed no general statistical support for a regressor sensitive to the interaction between past choice and past reward, as would be reflected, for instance, in win-stay, lose-shift behavior. We implement the perseveration regressor as an exponentially weighted sum over all past trials. We found that weighting previous trials with an exponentially decaying filter with a smoothing factor of 0.25 worked best (although slightly different parameter settings have almost equal cross-validation performance). Thus, we compute this feature on session *n* and trial *m* as such

18 $$\frac{1}{Z}\mathop{\sum }\limits_{k=1}^{m-1}\exp (-0.25\ k)\ (2\ {y}_{n,m-k}-1),$$

where $Z=\mathop{\sum }\nolimits_{k = 1}^{m-1}\exp (-0.25\ k)$ is a normalization constant, such that the entire exponential filter adds to 1. The transformation 2 *y* − 1 serves to encode responses as −1 and 1, for the purpose of having the perseverative feature sway the current response appropriately. Therefore, this feature reaches its maximal value of 1 if all previous responses were rightward and −1 if they were all leftward, putting it on the same scale as the other features. Timeout trials, where the animal did not respond before 60 s had passed, while skipped for the logistic regression of responses, are taken into account for the previous answer regressor, encoded as 0.

### Aggregation and interpretation of chains

We generally generated 48,000 samples from each of 16 chains (with different starting points), discarding the first 4,000 as burn-in. We assessed convergence of the chains using the classical measure $\hat{R}$^50^ and generated more samples by continuing each chain if necessary (although not all animals ever reached a sufficiently low $\hat{R}$ score, we excluded 12 animals for this reason). $\hat{R}$ compares intrachain and interchain variability of bespoke, state-independent features of the chains. To detect differences in the variances of the chains and other problems, which $\hat{R}$ is known to miss, we also used folded-$\hat{R}$ and rank-normalized-$\hat{R}$^51^. We reduced the memory cost by thinning the chain, using only every 25th sample (we did this purely for memory reasons, not because it is necessary for MCMC algorithms^52^). For a first pass, we sought to discard chains that differed substantially from other chains in the explored region in parameter space, either because they never reached the relevant parts of it or because they spent disproportionate amounts of time in some modes over others. This is a known problem for MCMC algorithms in multimodal environments and can be mitigated by taking nonmixed chains and combining them via stacking^53^. However, because our goal here is not prediction, we still want to focus on finding and visualizing the most important modes of the posterior, which we did by combining the (possibly not perfectly mixed) chains, and considering the regions of probability space in which they collectively spent the most time. Given the slow transitions between different modes, we also did not split our individual MCMC chains when computing $\hat{R}$, as the two halves of the chains were often too different.

As scalars underlying $\hat{R}$, we used the concentration parameters *α* and *γ*, as they are independent of states. We also included general properties of the fit—the number of trials assigned to the state with the most trials, the second-most trials and the overall numbers of states with more than 20% and more than 10% of trials assigned to them (we chose multiple cutoffs to gain information about the fit at different levels of resolution). By greedily discarding the chains that increase $\hat{R}$ the most, we reduced the number of chains under consideration from 16 to at least 8. For this, we considered all features and all variants of $\hat{R}$ (normal, folded, rank-normalized) at once, so we were minimizing the maximum over all these $\hat{R} {\rm{s}}$. We only further processed the chains when $\hat{R} < 1.05$, which is more conservative than some recommendations, but, in light of the strong multimodality, more lenient than the newest ones^51^.

However, it is still not trivial to extract information from the remaining chains given the multimodality. There are two main sources of multimodality, which are as follows: (1) genuine uncertainty in the usage of states or the exact setup of the random variables of the states, and (2) mode equivalence with permuted labels (for example, state *i* = 1 in the first chain might explain roughly the same set of trials as state *i* = 2 in the second). Although the second source makes evaluating the results more complicated, it is in fact just the sampling scheme working correctly, as there is nothing special about the particular state labels—solutions with permuted state labels are functionally equivalent. For the same reason, even within a single chain, a relatively consistent set of trials might be explained by one label for some part of the chain, but by a different label in another. Indeed, we frequently observed this kind of label switching, where one state completely took over the trials of another within a few sampling steps. In the limit of infinitely many samples, we can expect any trial to have a uniform distribution over the state label assigned to it; the only important question is which other trials were usually accounted for by the same state as the given trial within suitably similar samples.

To formalize the necessary abstraction from direct state assignments, we computed co-occupancy matrices *C*^*j*^ for each sample *j*. *C*^*j*^ is a matrix of size *T* × *T*, with *T* being the total number of trials across all sessions of a mouse, whose *t*, *m*th entry reports whether trials *t* and *m* (for convenience, dropping the additional session label) used the same state in sample *j*

19 $${C}_{t,m}^{j}={\mathbb{1}}({x}_{t}={x}_{m}).$$

We used these co-occupancy matrices as a basis for the following two different processing steps: (1) at a coarser resolution across trials, we applied dimensionality reduction to find posterior modes; (2) at full resolution, we averaged *C*^*j*^ across similar samples *j* to derive a matrix that describes the mutual affiliation of trials, allowing us to overcome the labeling issues. Both steps are reminiscent of representational similarity analysis^54^, in that, instead of comparing two samples directly, we compare state co-occurrence within the samples.

In principle, to explore the posterior, we could have flattened each *C*^*j*^ into a **T**^2^ vector and applied principal components analysis (PCA). However, there were too many trials per mouse (of the order of 15,000) to do this at full resolution, so we binned the trials into 200 bins, ignoring session boundaries, and then used the Wasserstein distance to measure state co-occurrence between the bins. That is, we define modified matrices ${C}^{{\prime} j}$ as

20 $${C}_{t,m}^{{\prime} j}=\mathop{\sum }\limits_{i=1}^{L}1-| {p}_{t,i}^{j}-{p}_{m,i}^{j}|,$$

where ${p}_{t,i}^{j}$ is the proportion of trials in bin *t*, which is assigned to state *i* in sample *j*. ${C}^{{\prime} j}$ reduces to *C*^*j*^ for bins comprising a single trial. We then plotted individual samples in the first three dimensions of the PCA space arising from flattened versions of ${C}^{{\prime} j}$, as shown in Supplementary Fig. 4.

In doing this, we found that the posterior for a number of animals wanders itinerantly between different modes, reflecting true uncertainty. These modes are distinct solutions and should not be blended. To isolate them, we performed Gaussian density estimation in the 3D PCA space to identify the ones that were most prevalent, as the regions of highest estimated density. We used this clustering to select samples $j\in {{\mathcal{J}}}^{\eta }$ that were sufficiently similar as to comprise an individual mode ${{\mathcal{J}}}^{\eta }$. For now, we did this by hand; however, the process could be made more formal by fitting a mixture of Gaussians to the posterior and then selecting samples around the means of the Gaussians with sufficiently large mixture weights. We selected at least 400 samples from a mode to form a representative collection.

Next, we sought to understand how trials within that mode were co-assigned to states. To do this, we averaged the co-occurrences ${C}^{\eta }=\frac{1}{| {{\mathcal{J}}}^{\eta }| }{\sum }_{j\in {{\mathcal{J}}}^{\eta }}{C}^{j}$ and treated ${\tilde{C}}^{\eta }=1-{C}^{\eta }$ as a distance matrix, where trials were close if they shared a state in most samples in the mode. We then performed hierarchical clustering on ${\tilde{C}}^{\eta }$, using as a cluster distance $d(\upsilon ,\nu )=\max({\tilde{C}}_{\upsilon [k],\nu [l]}^{\eta }),k\in \upsilon ;l\in \nu$, which took as the distance between clusters the maximum distance between any two trials in the clusters *υ* and *ν*. The result of the hierarchical clustering was a tree on the individual trials; cutting this tree at a certain level leads to a specific clustering. Thus, cutting at, say, 0.6 means that we only have clusters in which every trial was explained by the same state in at least 40% (1 − 0.6) of the samples. For our plots, we cut at 0.95, which empirically returned good results. Although this meant that trials needed to use the same state in only 5% of samples to be in one cluster, most trials were assigned to the same state much more frequently (Supplementary Fig. 5). This also shows a number of alternative clusterings from different thresholds, demonstrating that there is little change across a wide range of thresholds—the 95% threshold leads to 8 states with 100% trial coverage, an 80% cutoff leads to 9 states and 99.92% coverage, a 50% cutoff gives 12 states with 98.77% coverage and, finally, a threshold at 20% gives 15 states and 95.27% coverage. We can thus see that low criteria led to trials becoming unassigned and some states splitting apart, which is why we chose a rather high cutoff. A further verification that the procedure and its threshold gave a faithful representation of the collection of samples comes from comparing the overall solution against individual solutions from single samples. Empirically, these did indeed align. Our later recovery analyses also used this approach.

The states we show are therefore defined at heart by sets of trials. To compute the PMFs of such a set, we first considered a single MCMC sample and noted which states it assigns to the trials within this set on a session-by-session basis (although each individual trial only had one state assigned in a single sample, for the whole set of trials, it usually will not just have been a single state, due to random fluctuations, but mostly a single state). We turned the psychometric weights of these states into PMFs, over which we then averaged (in a weighted manner, considering how often any state occurred in the set of trials). For a single sample, this resulted in an average PMF of that state for each session. This then got averaged across samples within a cluster (evenly over all selected samples of a mode) to obtain the ultimate PMFs of this state.

To determine how closely a single trial is connected to its assigned state, we averaged the proportions of samples in which it was in the same state as all the other trials assigned to this state. That is, for a given trial *t*, we took a row of the consistency matrix ${C}_{t}^{\eta }$ and considered only the entries corresponding to other trials within the state under consideration. We then averaged over those entries, yielding the average proportion of co-assignment. We think of this as a proxy of the posterior over which state a trial is assigned to, and we show it in Fig. 3.

### Psychometric type classification

We observed by eye that the PMFs that the model found for the behavioral states had a tendency to fall into one of the following three characteristic classes: flat (type 1), half-tuned (type 2) and fully tuned (type 3). However, the boundaries between the classes were blurry, so we sought an objective distinction, recognizing its inevitable arbitrariness. Note that a state may change its type through the slow process; it is thus a session-dependent classification.

The measure we used in the main paper is the mean reward rate implied by the PMF on easy trials (100% and 50%), ignoring the effects of perseveration (and the debiasing protocol). We chose the reward rate because this tends to grow as the animals proceed from ignorance to competence. We chose to assess only the easy trials because early PMFs were not defined on the lower contrasts (because these stimuli were not presented), and including more difficult contrasts can lead to lower reward rates for more broadly defined PMFs, even when they are better on easy contrasts. Supplementary Fig. 6 shows the distribution of such reward rates across all states. It is apparent that there is a rather clear grouping of PMFs with reward rates below 0.6, defining type 1. The boundary between types 2 and 3 is somewhat less evident, implying that edge cases will be hard to assign. The threshold reward rate of 0.78 served reasonably well, as evidenced in Fig. 4. We further split type 2 PMFs on whether they were left-biased, right-biased or symmetric. We considered a PMF symmetric if its error rate on 100% leftwards contrasts was within 10 percentage points of the error rate on 100% rightwards contrasts.

### Cross-validation and ablations

Our model contains a number of free parameters that we set using a cross-validation procedure. We used this most notably for the variance *σ* of the normal distribution over how much the logistic weights of a state can change from session to session and the decay constant of the exponential filter over previous actions, which are fixed parameters that are not inferred during the inference procedure. This inference procedure is itself guided by priors, which we set to be vague, exerting minimal influence upon the ultimate posterior. However, their precise setting can nevertheless also be evaluated via cross-validation. This applies to the two gamma distributions over the concentration parameters *α* and *γ* and the priors over the parameters of the states’ duration distributions. Cross-validation also allowed us to verify that our usage of the weak-limit *L* = 15 did not hurt our model fits, and that including a win-stay lose-switch feature, indicating which side was or would have been rewarded on the previous trial, was not beneficial in capturing animal choices during learning.

We used a tenfold cross-validation scheme, randomly masking 10% of trials on each session. Because we were not interested in the details of the fits, we only ran one chain of 10,000 samples for each parameter combination and cross-validation fold we wanted to test and evaluated the quality of the fit through the summed negative log-likelihood on the last 4,000 samples on the held-out trials, which was sufficient for a stable estimation of the held-out log-likelihood. Despite this time-saving strategy, there were too many combinations of parameters to check exhaustively, so we used a manual heuristic search over promising combinations, finding an optimal setting and verifying that any relevant deviations from it only lowered the negative log-likelihood (Supplementary Fig. 7, left). As another measure to save computation, we only evaluated two folds of each animal for each parameter setting, but because we evaluated our model on 154 mice (this was before exclusions due to missing sessions or too low $\hat{R}$), we still evaluated on a substantial number of folds in total.

We tested the perseveration decay constant over the set of values (0.15, 0.2, 0.25, 0.3, 0.35, 0.4), the variance *σ* over the set (0.01, 0.02, 0.03, 0.04, 0.06, 0.12, 0.24), representing the small range that we found desirable for a consistent state identity, as well as some larger values to ensure that they did not outperform smaller variances. The search also included a larger support for the *r* parameter of the duration distribution (running from *r* = 2 to *r* = 905) and different settings of the *α* and *γ* concentration priors, which were independently varied over the set ((0.1, 0.1), (0.01, 0.01), (0.001, 0.001)).

Many of the parameter configurations yielded comparably high performance. Of note, the parameter setup closest to the selected model simply allows higher *r* values in the duration distribution, representing a strict extension of the model that, however, does not improve fit. When studying the correlations across two different parameter settings, but within the same animal and the same cross-validation fold, we found extremely strong correlations, with only slight offsets from the identity line and a small handful of outliers accounting for the differences. This provided evidence that the fits were fundamentally the same, and different mice did not significantly benefit from different settings, allowing us to simply take the best among many good settings and proceed with it for the population-wide fit. These settings were the ones specified throughout the study—perseveration = 0.25, *σ* = 0.04, *r*_*i*_ ~ *U*(5, 6, 7, …, 704) and both *α* and *γ* ~ Gamma (0.01, 0.01).

In addition to finding the best parameters for our fit, we also used this approach to ablate the most important model components, verifying that all aspects of the model were necessary to provide as good a fit as possible within our framework (Supplementary Fig. 7, right). In particular, we tested the best parameter setting we found, but did not allow for change in weights between sessions (effectively removing the slow process of the model), both with 3 states (thus emulating the work described in ref. ^21^, although with duration distributions) and with the usual upper bound of 15 states. Allowing for 15 states but no slow process led to only somewhat worse performance than the full model (Supplementary Fig. 7, right—‘15 states, no slow proc.’), but did so at the cost of significantly increasing the usage of short-lived states. We tested this by considering how many states explained more than *x*% of trials of an individual animal (which can be read directly from the cross-validation samples, not requiring the sample aggregation procedure described previously). The full model makes more use of highly prevalent states that explain more than 20% of trials—1.7 ± 0.53 (mean ± s.d.) per animal versus 1.07 ± 0.67 of such states for a model without the slow process (two-sided Mann–Whitney *U* test, *U* = 21858.5, *P* < 1 × 10^−30^, effect size = 1.18 (standardized mean difference with s.d. over full model state number), *n* = 154 mice), but fewer overall states, such as any that explain more than 2% of trials—5.16 ± 1.62 versus 9.13 ± 2.14 (two-sided Mann–Whitney *U* test, *U* = 87773.5, *P* < 1 × 10^−73^, effect size = 2.45, *n* = 154 mice). Thus, while the removal of the slow process can mostly be made up for by an increased reliance on new states (for which our model has plenty of capacity), the slow process benefits the fits by tying together highly similar trials across short timescales, rather than arbitrarily separating them when behavior gradually changes too much to be accommodated by a single state.

We also allowed only one state (including the slow process), removing the notion of multiple states from the fit (Supplementary Fig. 7, right—‘1 state’). This model performed, perhaps surprisingly well, but because a session is usually dominated by a single state, a single adaptable state may perform somewhat well. We tested whether a win-stay lose-switch (WSLS) feature, indicating which choice was or would have been rewarded on the last trial, was beneficial, which it was not (Supplementary Fig. 7, right—‘Best + WSLS’), and whether the perseveration feature could be removed, which it could not (labeled ‘No perseveration’). Finally, we also tested the improvement due to the duration distributions (which replaced the implicit geometric duration distribution of an HMM; Supplementary Fig. 7, right, ‘No duration (exp. only)’). This test proved somewhat problematic within our framework, as restricting the model to implement durations through the transition matrix led many of the posteriors to settle on an unsatisfying solution. In this solution, states were extremely strongly biased leftwards or rightwards and rapidly alternated, depending on the choice of the animal. Such a model has, of course, almost no predictive power on held-out trials. This is seemingly a consequence of the hierarchical nature of the transition matrix—if we often transition into a state (and without duration distributions, we have a state transition after every single trial, with most of them being self-transitions), it becomes generally attractive in the iHMM framework, encouraging transition distributions that are much closer to uniform than one would expect for a reasonable notion of temporally extended states. We thus implemented geometric distributions that prefer longer states by fixing *r* = 1, but biasing the prior over *p*. We performed another small cross-validation sweep and present here the best model found in this way.

### Posterior predictive checks

To identify any mismatches between our modeling assumptions and actual behavior, we performed posterior predictive checks using multiple test statistics. The goal of this analysis was to determine whether responses generated solely from posterior samples reproduced the behavioral trends observed in the actual data. We simulated behavior for each session of an animal by taking each sample from our selected posterior mode, initializing with the state that was the actual state on the first trial for that sample and then generating responses. We needed to initialize with the true state, because the model uses a static initial state distribution **π**_0_, so a random initialization would lead to an unstructured mix of proficient and inexperienced behavior. However, after initializing the first state, the model ran completely independently—we drew a duration from the duration distribution of that state, using posterior parameters, randomly sampled a next state from the transition matrix once a state ended and sampled responses from the observation distribution of the current state, given the current features. These features included the contrast that was presented on that trial and a recomputed perseveration feature based on the choices of the current run of the simulation (so notably not the perseveration feature based on the choices of the animal). This unguided generation of behavior thus represents a very stringent test of the posterior fit.

We visualized the results by plotting actual behavior in relation to the distribution created by simulating behavior three separate times with each sample (because we use at least 400 samples from a mode, this equates to >1,200 simulations). As metrics of interest, we chose the percentage of correct choices in a session and the percentage of rightward choices for each contrast. We plot the accuracy of a single individual (the mouse of Fig. 2) in Supplementary Fig. 8a and the PMF on the last session of that animal in Supplementary Fig. 8b. As we can see here, behavior simulated from the posterior generally provides both a tight as well as accurate estimate around the true behavior.

To summarize the relationship between true behavior and the simulated distribution across the population, we calculated the percentiles of the empirical values within the simulated distribution, visualized in Supplementary Fig. 8c,d. In an ideal case, the histograms over these percentiles would be uniform, indicating that the posterior provides an unbiased and calibrated estimate for the true behavior. This is not quite true here—we can see that accuracy has a modest tendency to be overestimated (that is, the true accuracy tends to fall onto lower percentiles of the simulated distribution). As mentioned in the ‘Discussion’, behavior often degrades toward the end of a session (almost by necessity, as it is one of the session termination criteria), but this was not always acknowledged with a separate state by the model, perhaps because behavior degrades in a gradual and inconsistent manner across sessions. We suggest this as an interesting direction for a possible extension of our framework, by combining the states with a mechanism for change on a shorter time scale, similar to the work described in ref. ^28^. However, implementing this in a way that keeps states distinct and has them retain their identity over long time periods seems challenging, in the face of motivational changes that occur gradually but can change behavior quite notably on the order of tens of trials. Note that the overestimation of accuracy also occurs on sessions on which the model does ultimately include a state that reflects a substantial reduction in performance. This happens because the model sometimes fails to appropriately transition to this worse state (given that it is only a descriptive model with no foresight of when a session ends). Thus, accuracy in free simulations can be too great.

While the percentage of rightwards choices across contrasts forms a seemingly uniform distribution, splitting the histogram over the different contrasts reveals that there is a modeling assumption that biases the estimates for the different contrasts somewhat, as shown in Supplementary Fig. 8e. Most notably, for the 100% contrasts, the model underestimates how accurate the animals are (by overestimating the % rightwards choices on leftwards contrasts and vice versa). Note, however, that the insets for these contrasts show that the actual deviation is very small. Somewhat more subtly, the opposite occurs for the respective 50% contrasts. These deviations arise from the psychophysical transform we borrowed from refs. ^21,28^, namely the tanh transformation on the raw contrast values. The 100% and 50% contrasts are mapped onto very similar values (1 and 0.987, respectively), strongly coupling the percentage of rightward choices for the two contrasts, requiring them to take on almost the same value. This is intuitively desirable—allowing a smoothing over the different contrast strengths and reducing the number of parameters in our logistic regression (using a general ‘leftwards sensitivity’, rather than having a separate parameter for each contrast). While 100% and 50% are very different in terms of absolute value, they are both highly visible, meaning their difference from a psychophysical perspective is rather minor^55^. Nevertheless, as it turns out, some mice can occasionally exhibit rather different behavior on the two contrasts (Supplementary Fig. 8e, insets), leading to an underestimation for the stronger contrast and an overestimation on the weaker one.

The 0% contrast plot, on the other hand, exemplifies a posterior predictive check without such reservations—there is no noticeable bias, and the posteriors appear correctly calibrated. The predictive checks thus serve as an important tool to study the limitations of our modeling approach, highlighting that degrading behavior is not fully captured by the model and that the smoothing over contrasts imposes some structure onto the PMFs that biases the performance estimates. To study further the effect these biases in the model have upon the fits, we analyzed the magnitude of the bias imposed (Supplementary Fig. 16). As we can see, most of the differences fall within a close range around the posterior mean.

As a proof of concept, we refitted the model with a different PMF parameterization to see whether this could address the observed issue. This alternative parameterization was inspired by another line of work that uses neural networks to capture animal behavior on the IBL task. Using this, we mapped the contrast strengths (1, 0.5, 0.25, 0.125, 0.0625, 0) onto (1, 0.899, 0.705, 0.416, 0.207, 0) for the logistic regression, whereas the tanh transformation mapped onto (1, 0.987, 0.848, 0.555, 0.302, 0). The results of repeating the posterior predictive checks on a representative random sample of mice (*n* = 84) using this new parameterization are shown in Supplementary Fig. 9. This reveals that the tension between the predictive distributions on 100% and 50% contrasts was mostly caused by the PMF parameterization, rather than by the model itself. Because the fits under this new PMF did not qualitatively differ from fits under the old parameterization, we did not redo our analyses, but accepted this as evidence for the suitability of the model and fitting procedure. The remaining slight tension between the predictions on strong contrasts might be caused by changes in the perceptual sensitivities of the animals during learning, which is an interesting avenue to pursue in further studies of learning.

### Model recovery

We tested the model and our inference procedures by fitting to data for which the ground truth was available. For this, we instantiated all the random variables of the model to specific values and generated responses from it. This was performed for multiple different variable settings to assess the accuracy of the fitting procedure in all relevant regimes and using input data (that is, contrast sequences) from actual training trajectories. The data generated this way were processed exactly as those from the IBL mice.

We paid particular attention to assessing the strength of the inductive biases of the inference procedure—particularly in terms of the number of states it inferred (given that this could be potentially unbounded, within our weak-limit approximation) and the degree of change between sessions (because slow and fast state changes could interact). We tested multiple settings in which all the data were actually generated from a single state, to test whether the model would incorrectly split behavior into multiple states. In one setting, the psychometric weights of the state stayed constant throughout all sessions. In another, the weights gradually evolved from poor performance to proficiency (at constant steps of a magnitude that corresponds to a variance of 0.0311; the variance of the fitting procedure was fixed to 0.03). Both fits recovered their ground truth successfully, explaining virtually all trials with a single state, as can be seen for the example of the changing state in Supplementary Fig. 10. We also tried a variation of the latter situation, in which the psychometric weights changed in (proportionally smaller) steps on every single trial, rather than all at once at a session boundary (as the model assumes). This, too, was recovered by the model with only one state (which we consider the best possible solution, given that the generative process was outside the model class).

We also successfully recovered settings from 2 to 9 states, with and without session-to-session variation on the weights, with strongly varying trial proportions between the different states (Supplementary Fig. 14) and of varying overall training lengths (particularly to test whether long training trajectories lead the model to impose fewer states, making more use of the slow process), as seen in Supplementary Fig. 11. The model was also tested on a setting with completely implausible PMFs, but with the added difficulty of having a larger number of states active within each session (Supplementary Fig. 15). This, too, was captured accurately. These successful recoveries suggest that the model can uncover states that truly correlate with distinct modes of animal behavior.

### Reporting summary

Further information on research design is available in the Nature Portfolio Reporting Summary linked to this article.

## Online content

Any methods, additional references, Nature Portfolio reporting summaries, source data, extended data, supplementary information, acknowledgements, peer review information; details of author contributions and competing interests; and statements of data and code availability are available at 10.1038/s41593-025-02130-x.

## Supplementary information

Supplementary Information Supplementary Results, Table 1, Figs. 1–16 and a full list of IBL members.

Reporting Summary

## Data Availability

Please follow the instructions at https://int-brain-lab.github.io/iblenv/notebooks_external/data_download.html to download the data used in this article. Our public code contains a script to download the dataset.

## Code availability

The code used for this analysis, as well as installation instructions for the necessary packages, can be found at https://github.com/SebastianBruijns/diHMM.
