# Supplementary material for: Infinite hidden Markov models can dissect the complexities of learning
Source: Nat Neurosci. 2025 Dec 30;29(1):186–94. doi: 10.1038/s41593-025-02130-x (PMC12779568; doi:10.1038/s41593-025-02130-x)
Supplement: Supplementary file 1 — Supplementary Results, Table 1, Figs. 1–16 and a full list of IBL members. [file 41593_2025_2130_MOESM1_ESM.pdf]

nature neuroscience

Supplementary information https://doi.org/10.1038/s41593-025-02130-x

Infinite hidden Markov models can dissect the complexities of learning

In the format provided by the authors and unedited

Supplementary Results

Bias training analysis

As elaborated, the learning stages exhibited by the animals seem rather independent, without strong patterns across the stages. This becomes even clearer when considering the next phase of learning, involving biased training: The basic task stayed the same, but instead of contrasts appearing equiprobably left or right, there were now unsignalled alternations between blocks, lasting 20-100 trials following a truncated exponential distribution, during which a contrast was 80% likely to appear on one side versus 20% on the other. This was of course particularly helpful for 0% contrasts, on which an animal could now reach a much higher reward rate than chance, given a suitable block inference mechanism (a detailed analysis of their actual algorithm was performed in Findling et al. (2023)). To finish this part of training, mice had to exhibit behaviour that was sufficiently modulated by the current block. The number of sessions it took them to achieve this was not correlated with the time spent in stage 1 or 2, although there was a slight negative correlation with the number of sessions in stage 3 (Correlation to stage 1 duration: Pearson's $r = -0.06, p = 0.47$; to stage 2 duration: Pearson's $r = -0.005, p = 0.96$; to stage 3 duration: Pearson's $r = -0.2, p = 0.02$, $n = 134$ mice). This suggests that learning about the biased blocks tapped into yet another type of skill, but extensive pre-training with the full contrast set gave some mice a slight edge here.

Findling, C., Hubert, F., Laboratory, I.B., Acerbi, L., Benson, B., Benson, J., Birman, D., Bonacchi, N., Carandini, M., Catarino, J.A., Chapuis, G.A., Churchland, A.K., Dan, Y., DeWitt, E.E., Engel, T.A., Fabbri, M., Faulkner, M., Fiete, I.R., Freitas-Silva, L., Gerçek, B., Harris, K.D., Häusser, M., Hofer, S.B., Hu, F., Huntenburg, J.M., Khanal, A., Krasniak, C., Langdon, C., Latham, P.E., Lau, P.Y.P., Mainen, Z., Meijer, G.T., Miska, N.J., Mrsic-Flogel, T.D., Noel, J.-P., Nylund, K., Pan-Vazquez, A., Paninski, L., Pillow, J., Rossant, C., Roth, N., Schaeffer, R., Schartner, M., Shi, Y., Socha, K.Z., Steinmetz, N.A., Svoboda, K., Tessereau, C., Urai, A.E., Wells, M.J., West, S.J., Whiteway, M.R., Winter, O., Witten, I.B., Zador, A., Dayan, P., Pouget, A.: Brain-wide representations of prior information in mouse decision-making. bioRxiv (2023) https://doi.org/10.1101/2023.07.04.547684 https://www.biorxiv.org/content/early/2023/07/05/2023.07.04.547684.1.full.pdf

Supplementary Figures

Quantity	Meaning	Counter
N	number of sessions	n
T_n	number of trials within a session n	t, m
J	number of MCMC samples	j
L	number of states in the model	i
S	number of states employed within a session	s

Supplementary Table 1: Overview over quantities and counters

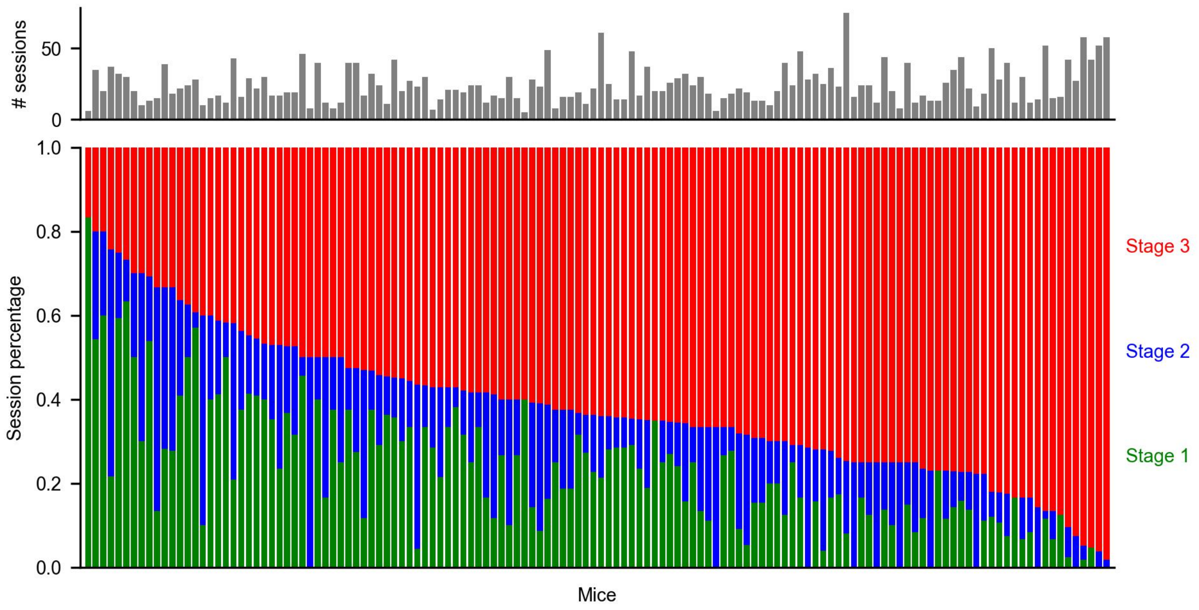

Supplementary Figure 1: The proportion of sessions per behavioural stage sorted by the proportion in Stage 3 **(bottom)**, with a histogram of the total number of sessions **(top)**. No strong trend between any of the stages or the total number of session emerges.

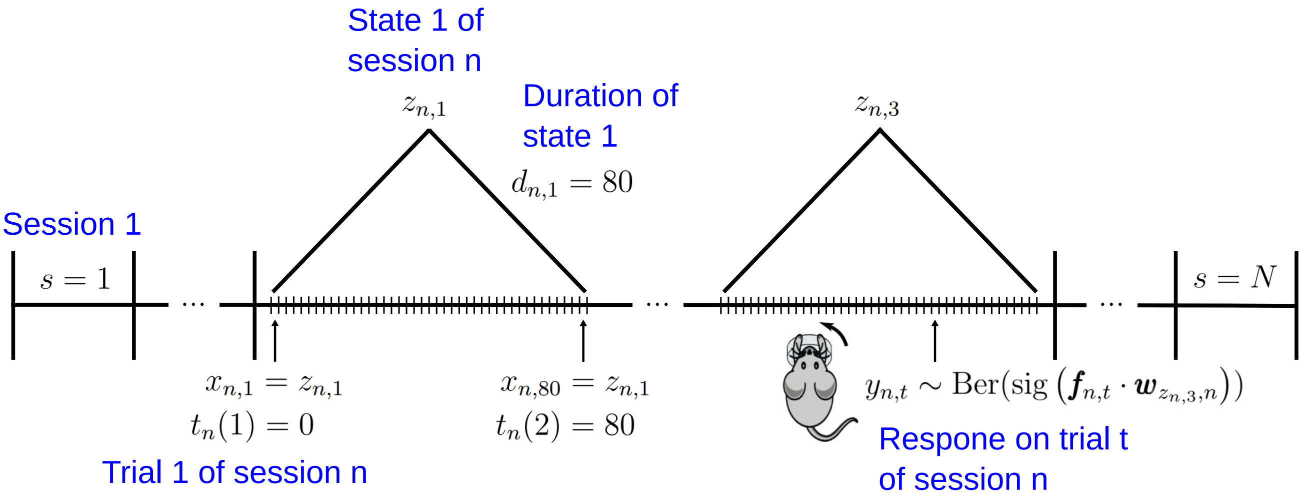

Supplementary Figure 2: Visualisation of the different variables across training, with some explanatory text in blue.

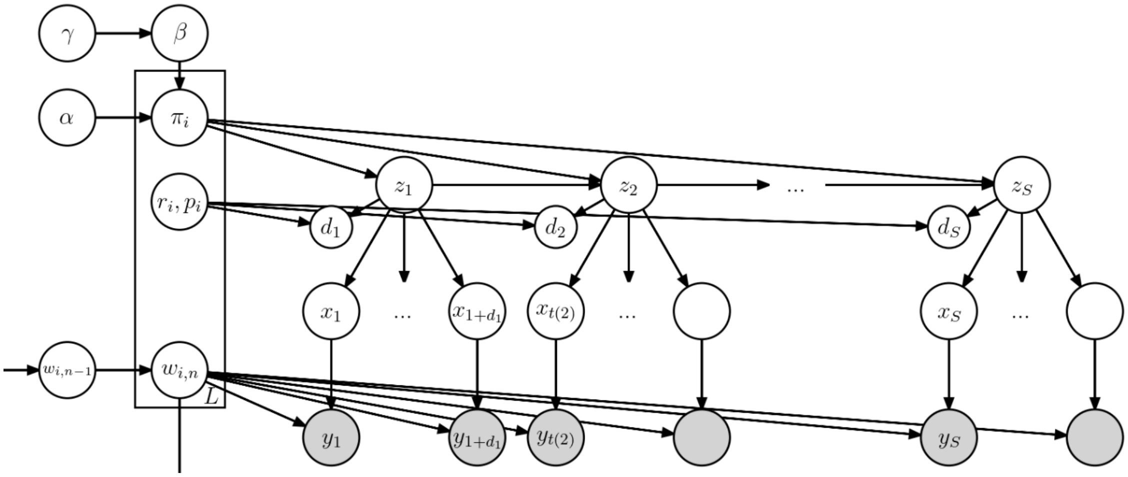

Supplementary Figure 3: Visualisation of the random variables as a graphical model, showing the variables z, d, x, and y of a specific session n. Variables in shaded circles are observed, unshaded variables are inferred. We suppressed the dependence on session n for all relevant variables other than the state weights w, for which we highlighted that they can indeed change across sessions.

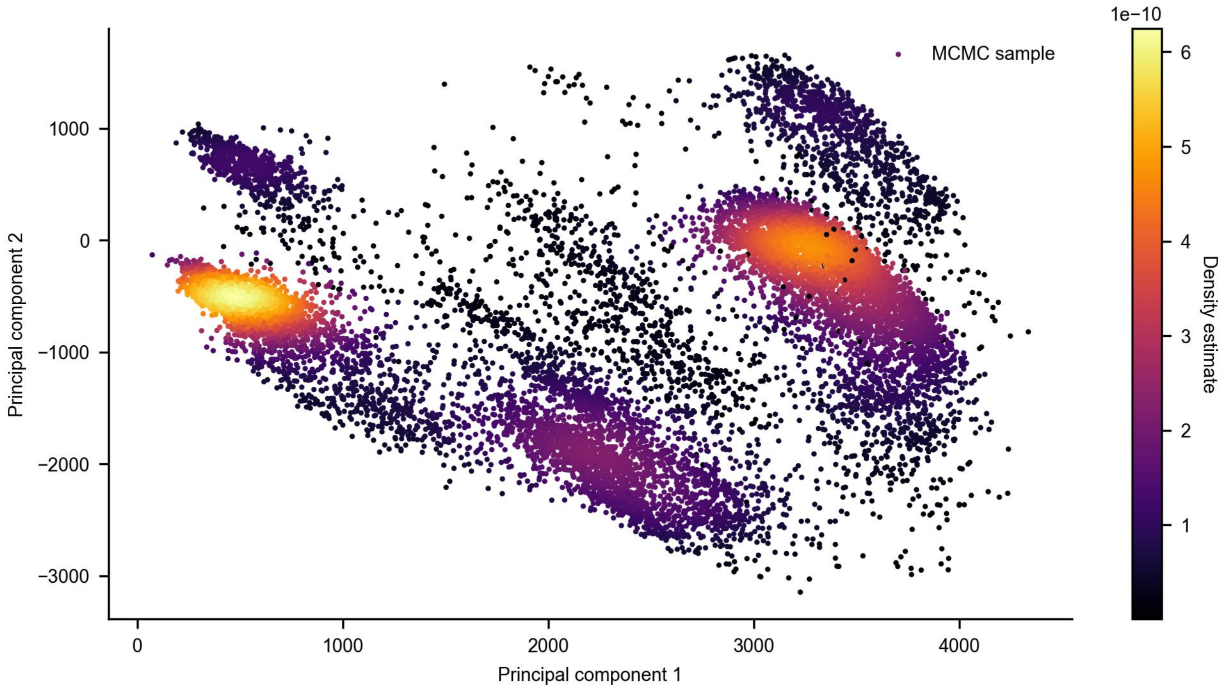

Supplementary Figure 4: Individual MCMC-samples can be scattered in 2D principal component (PC) space, to find regions of high probability. To make those regions more salient, we colour individual samples according to a Gaussian density estimation (the density estimation occurs in 3D PC space). There are multiple modes of varying importance, with one mode being particularly dominant.

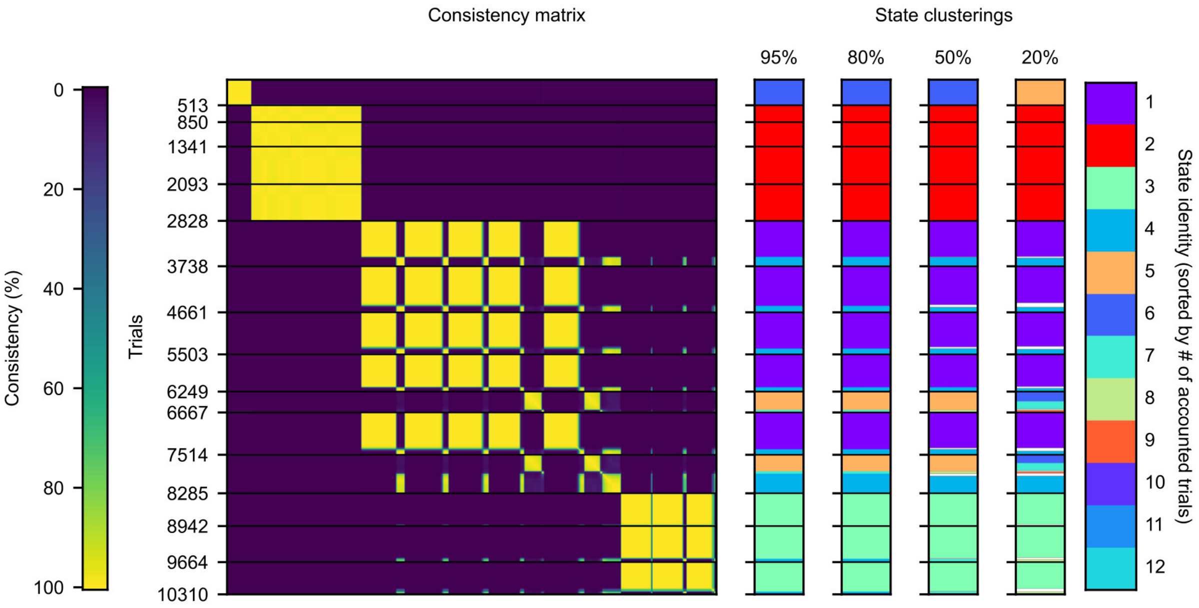

Supplementary Figure 5: Consistency matrix C^{η} of the animal seen in **Fig. 2**, with different state assignments on the right, based on cutting the hierarchical clustering tree at the different levels noted above the colouring assignments. The ticks on the left of the matrix mark session boundaries, with the numbers indicating the total number of trials so far. State colour on the bars to the right was determined based on the number of trials assigned to the state in a given clustering, therefore a colour change need not be a major change in state assignments. As can be seen, state assignments are robust in a large range of cutoff values, with large states staying particularly consistent. Most of the change comes from the splitting of smaller states, and some trials losing a state assignment altogether (a state needed at least 40 trials to be coloured; if the state of a trial had fewer than that, it is coloured white).

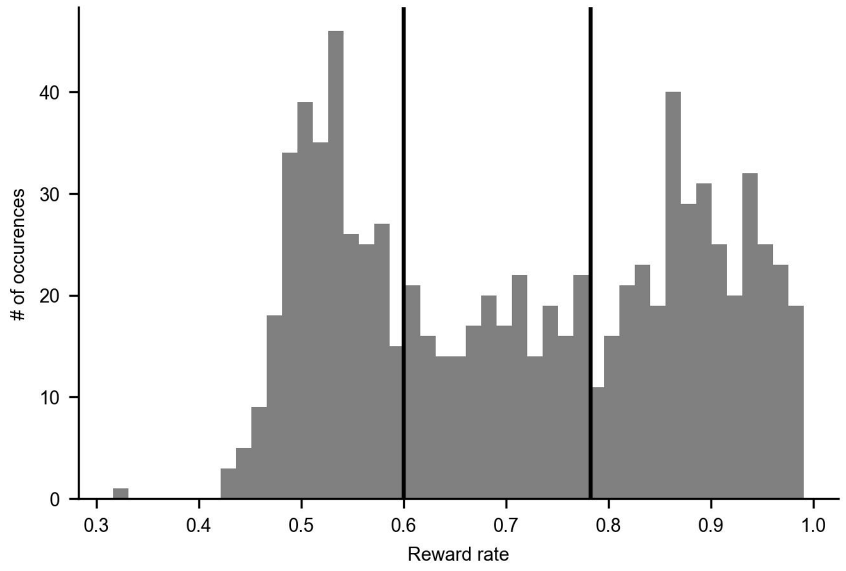

Supplementary Figure 6: Histogram of the mean reward rates on easy trials of the PMFs of all states, at the moment they first appeared. Vertical lines indicate the boundaries we used to classify states into the three types. The boundaries we drew do align with points of low density in the histogram.

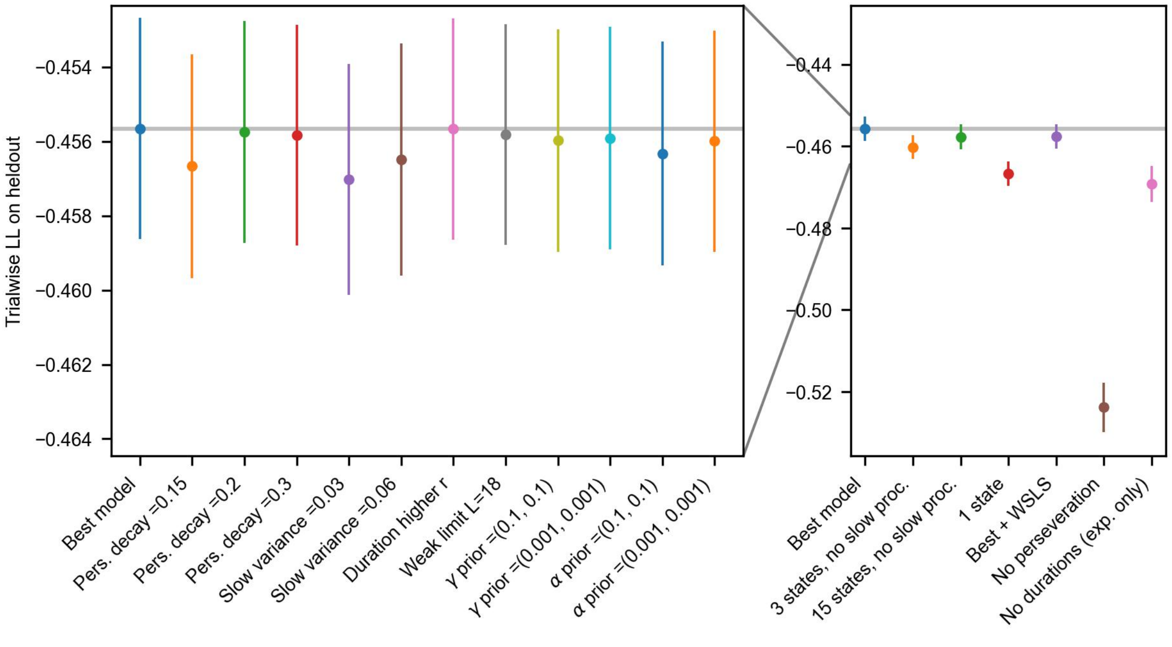

Supplementary Figure 7: Left, Cross-validation results over the best model, and all variants which represent a small change either way in one of the relevant model parameters (not all tested parameter combinations are depicted). Error bars represent 1 standard error of the mean ($n = 308$ cross-validation folds, from 154 mice (i.e. before exclusions) times two folds). The best model uses these parameters: perseveration $= 0.25$, $\sigma = 0.04$, $r_i \sim U(5, 6, 7, ..., 704)$, and both α and $\gamma \sim \text{Gamma}(0.01, 0.01)$. **Right,** Cross-validation results for ablated versions of the model. Note that the y-axis is considerably zoomed out from the plot on the left.

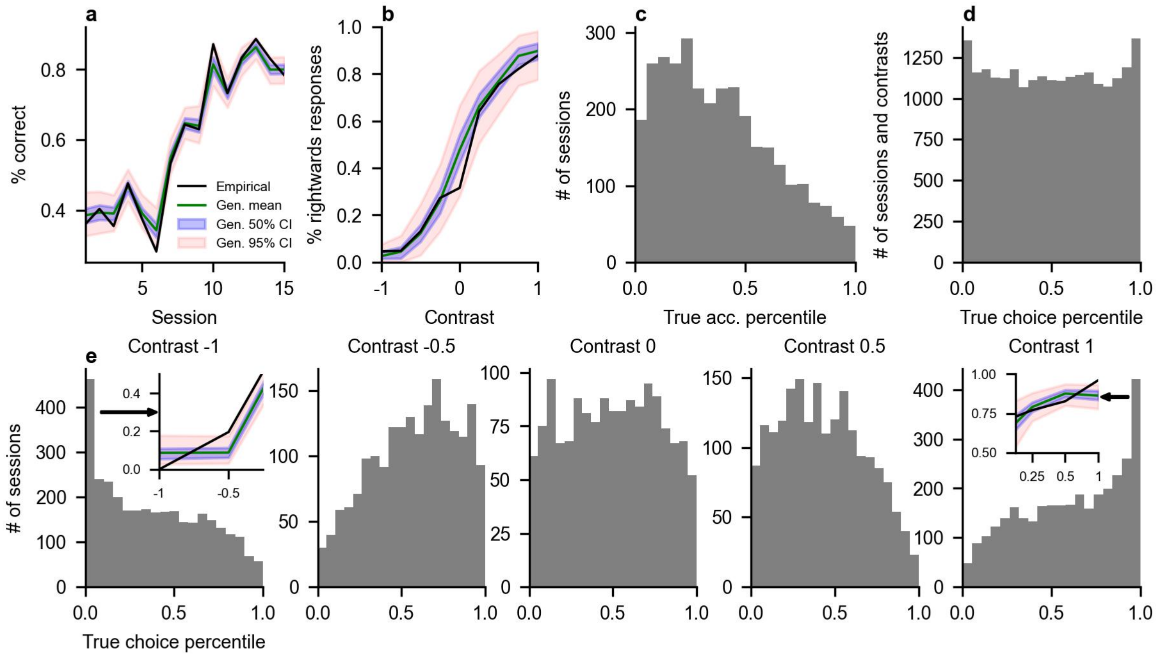

Supplementary Figure 8: Posterior predictive checks. **(a)** True session-wise accuracy of the animal shown in Fig. 2, together with the mean posterior accuracy, as well as 50% and 95% credible intervals, created through simulation. **(b)** The true PMF of the same animal on its last session, together with the posterior distribution over the PMF. Both accuracy and PMF are well captured by the posterior. **(c)** By computing the percentile onto which the true accuracy falls within the posterior for each session across all animals, we can visualise the posterior fit on a large scale. This reveals a tendency to overestimate the accuracy of the animal – see discussion in text. **(d)** We can do the same thing as in (c) for the PMF, now over sessions and contrasts. Here the posterior seems close to uniform, as is desired. **(e)** Splitting (d) by the different contrasts, however, reveals a bias that arises from the psychophysical transform we apply, which maps strong contrasts onto very similar values. This can lead to an underestimation of performance on the strongest contrasts, and an overestimation on weaker ones (see the insets, which show an example PMF as in panel (b), zoomed in on the relevant contrasts). When performance on these contrasts is quite different, as shown in the insets, the desired smoothing over behaviour can introduce biases – see further discussion in text.

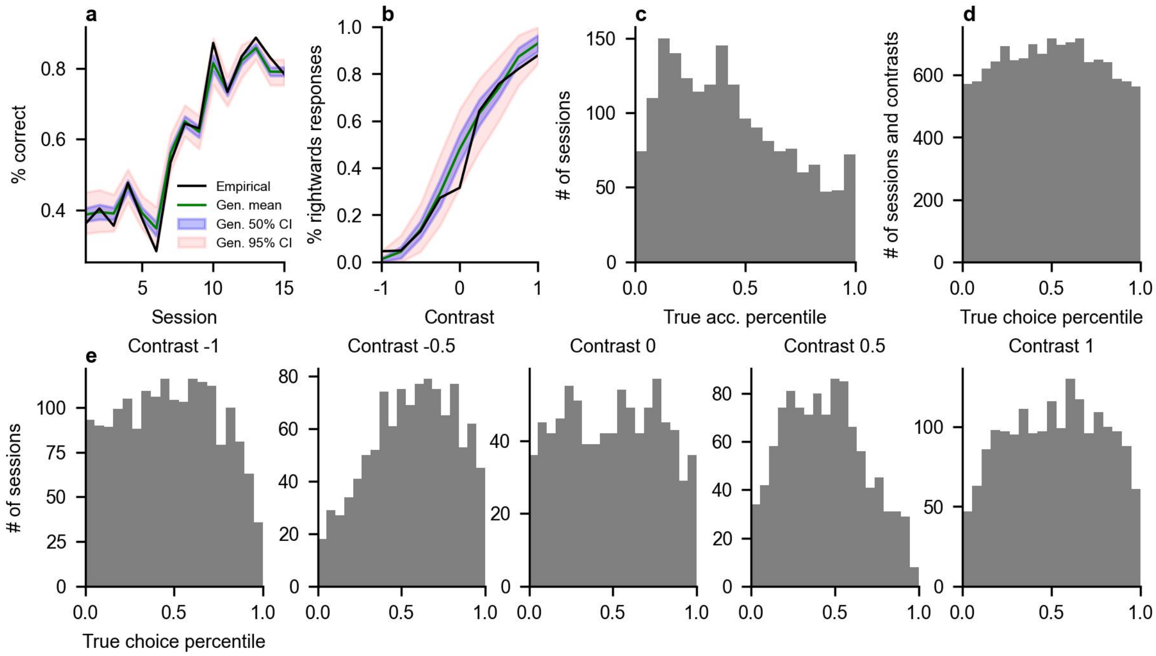

Supplementary Figure 9: Posterior predictive checks as in Supplementary Figure 8, but using a parameterisation of the PMF derived from a neural network. We tested this on a representative sample of $n = 84$ animals. This mostly removes the bias in predicting performance strong contrasts, showing that it is a question of parameterisation rather than the model itself.

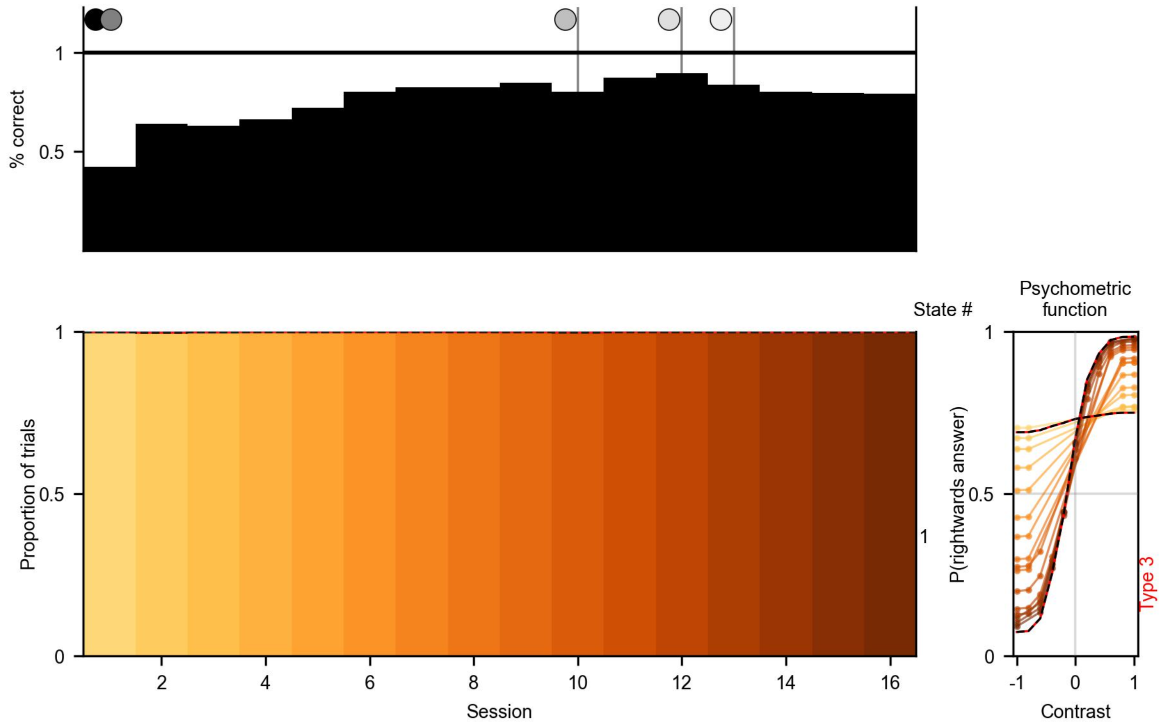

Supplementary Figure 10: Model recovery for a case in which behaviour was generated according to the model, using only one state with a changing PMF. The red and black outlines indicate ground truth, which was basically perfectly recovered. For the changing PMF, we only show the first and last ground truth (the initial PMFs are not incorrectly recovered for low contrasts, but are not uniquely identifiable due to the limited contrast set during this period)

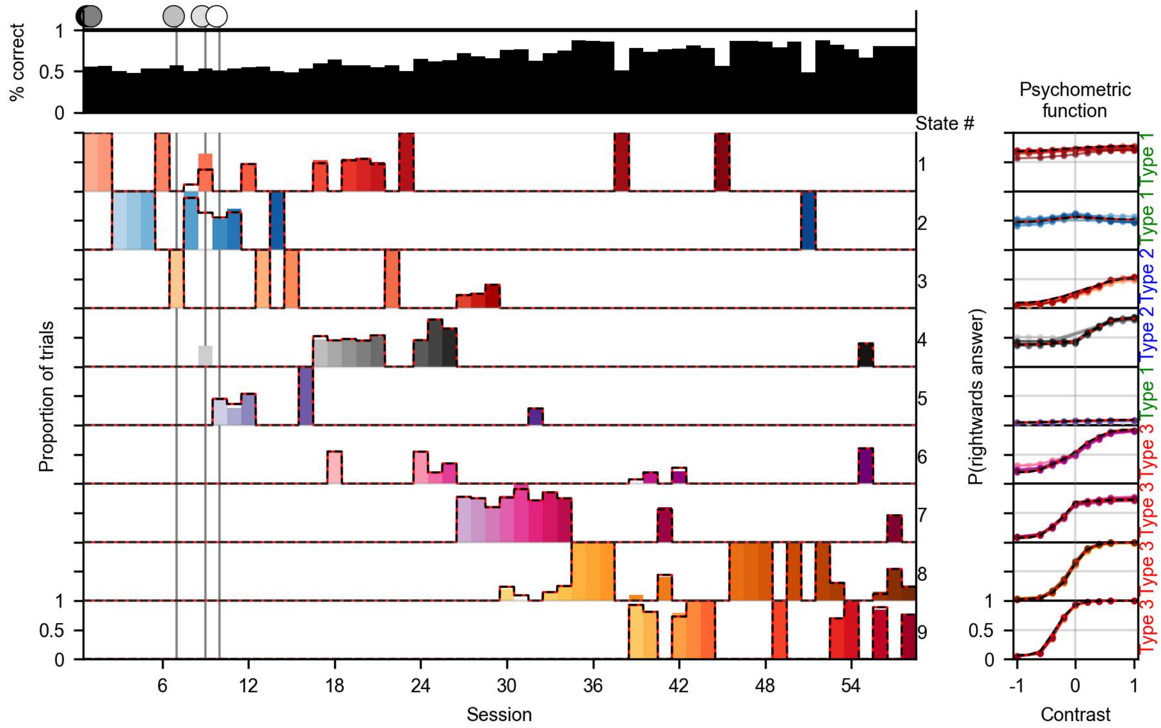

Supplementary Figure 11: Model recovery where behaviour was generated using 9 different states, on a large number of sessions. The red and black outlines indicate ground truth, which is recovered close to perfectly, in particular correctly recovering the number of states. On session 9, state 2 is incorrectly split between state 1 and 4, the only major flaw.

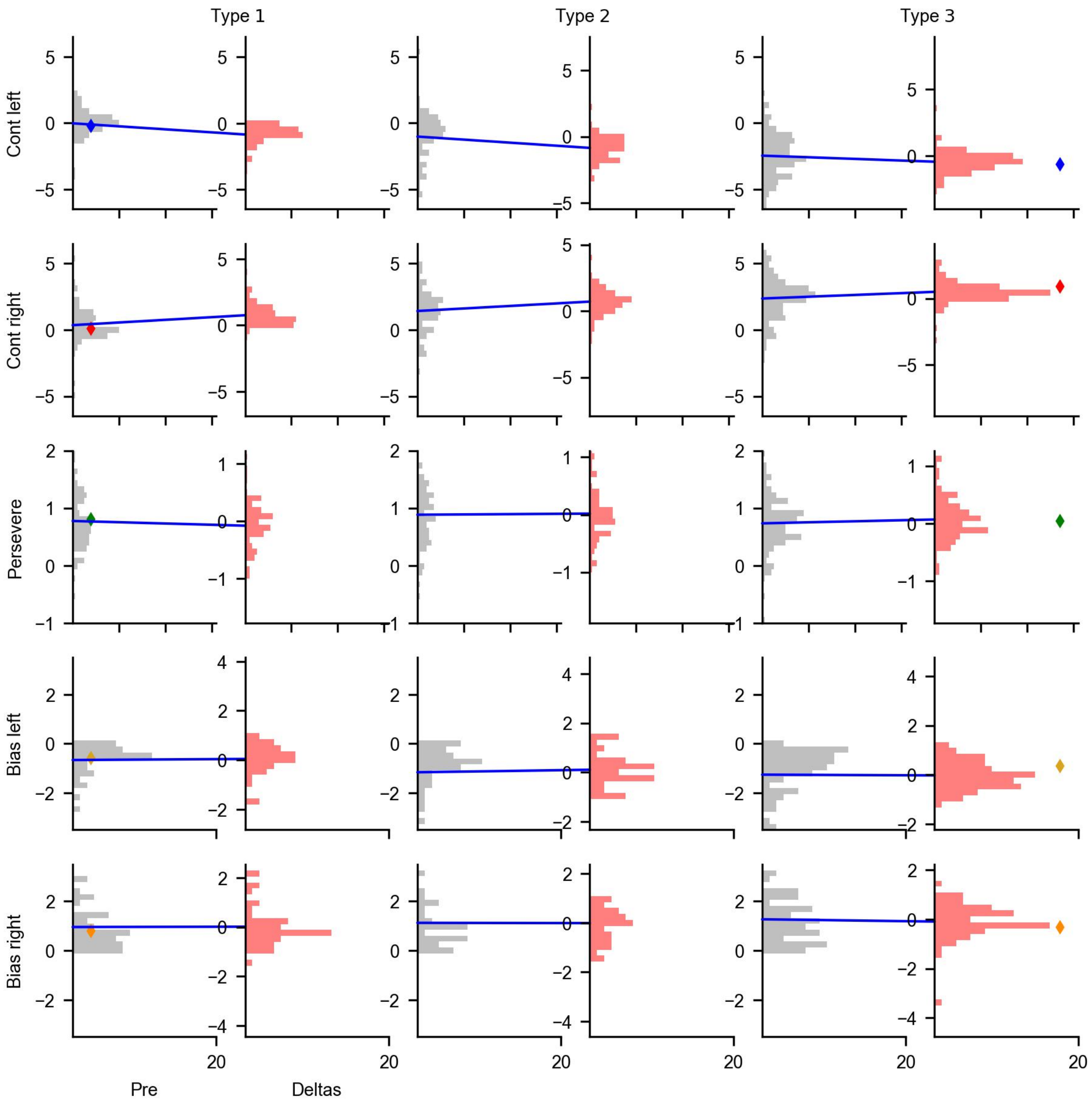

Supplementary Figure 12: Distributions of initial weights of states and how they changed across their lifespan, split by types. The red distributions to the right show the weight change, not the final distribution, to highlight the change through the slow change process. The blue lines connect the means of two related distributions (we use the mean of the initial distribution as the 0 point of the delta distributions). The bias is split as in **Fig. 6**, and the x-axis is shortened due to this, though the x-ticks are at the same distances across all plots. The coloured diamond markers denote the average weight of the first and last state of every animal, as in **Fig. 6**

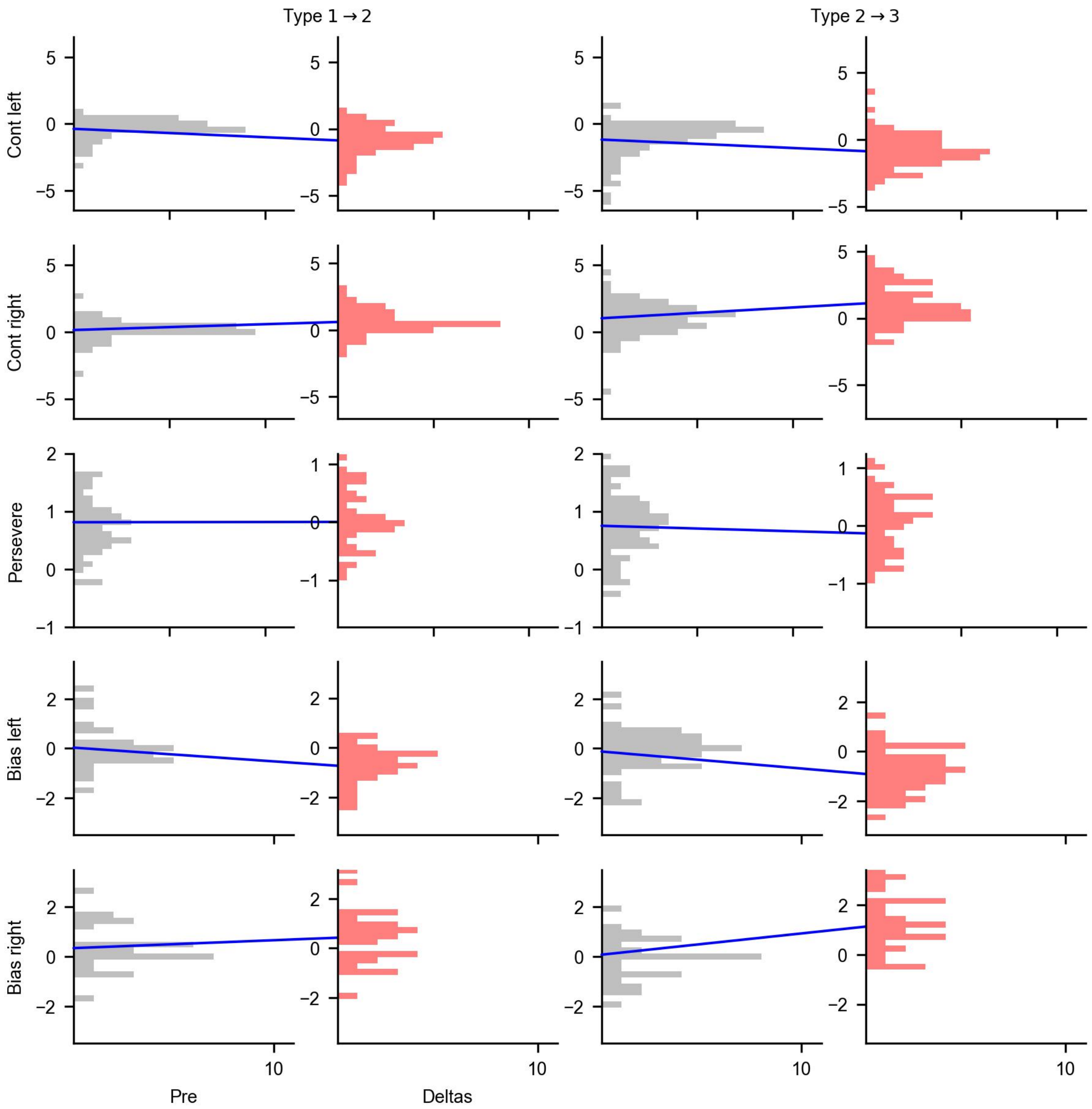

Supplementary Figure 13: Distributions of weights of the closest previous states and how they differed in the first introduced state of a new type. The red distributions to the right show the weight difference, not the final distribution, to highlight the change through the fast process. The blue lines connect the means of two related distributions (we use the mean of the previous distribution as the 0 point of the delta distributions). The bias is split as in **Fig. 6**, and the x-axis is shortened due to this, though the x-ticks are at the same distances across all plots.

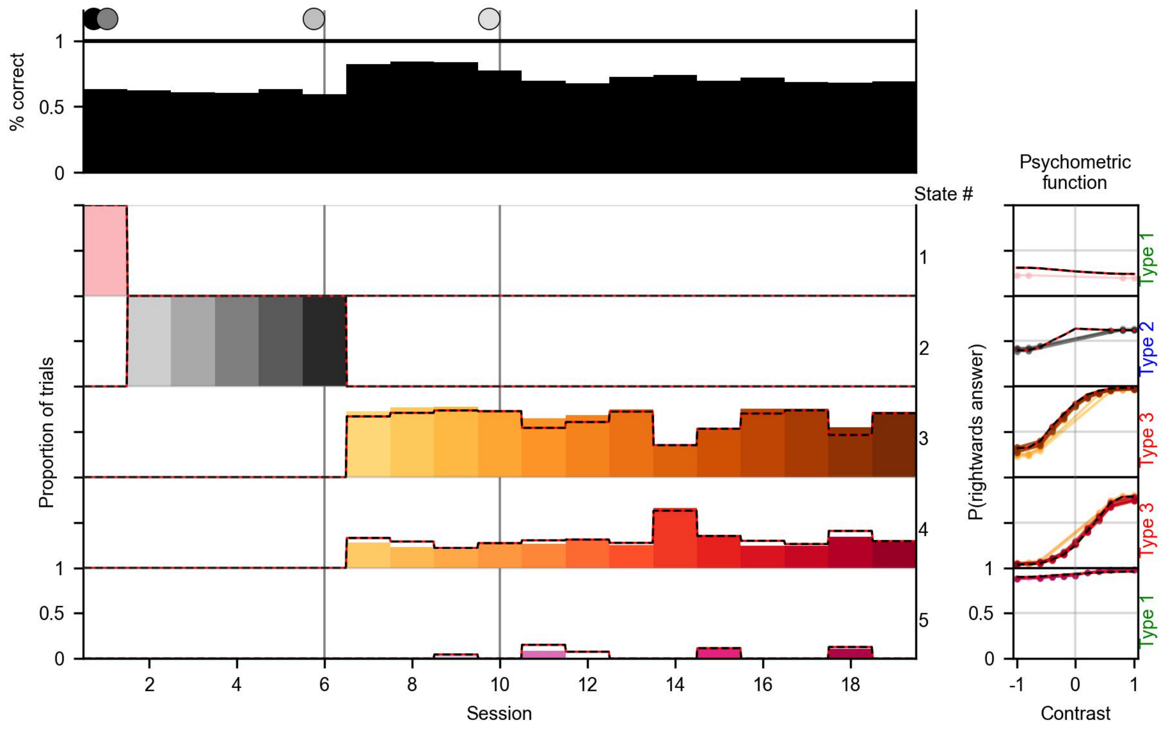

Supplementary Figure 14: Model recovery of 5 states in a typical progression, the red and black outlines indicate ground truth. This example shows the model's ability to distinguish between similar states (3 and 4) within a session. A small number of trials were incorrectly assigned between states 3, 4, and 5, in particular the rare state 5 missed some trials.

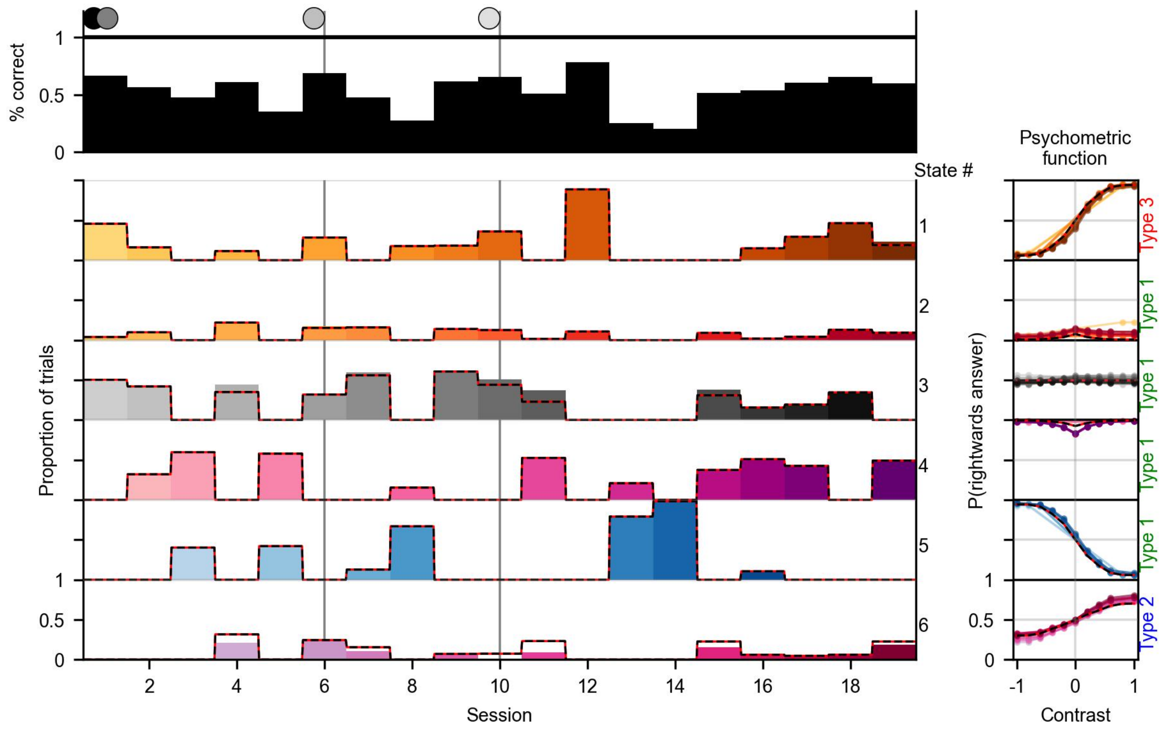

Supplementary Figure 15: Model recovery of 6 states with some unusual PMFs, which made the states more distinguishable, but the recovery is made more difficult by the fact that many states co-occurred in single sessions. The red and black outlines indicate ground truth. The model found the correct number of states for this recovery and almost flawlessly discovered the boundaries between the states in the sessions. Only trials of state 3 and 6, which were the most similar (noting that state 3 had the highest variance in its responses), were sometimes noticeably misplaced.

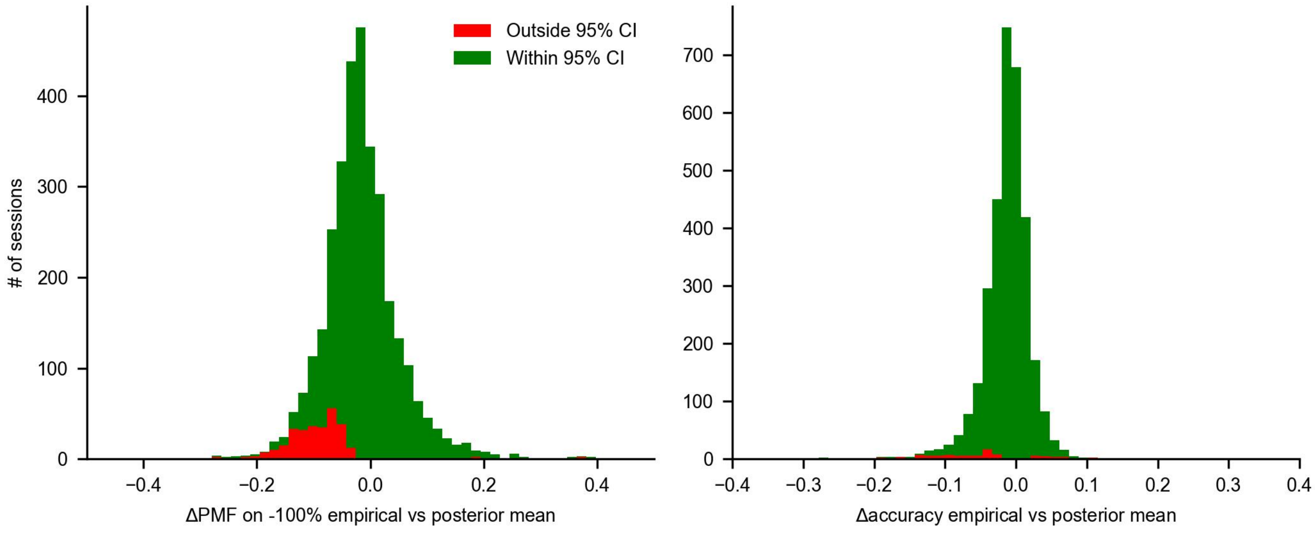

Supplementary Figure 16: To examine the biases evident in the posterior predictive checks, we plot the distance between the posterior mean and the empirically observed PMF on -100% contrasts **(left)**, and overall accuracy **(right)**. The green part of the histogram shows sessions which fall within 95% of the credible interval of the posterior, and red those that fall outside of it. Note that we expect 5% of sessions to fall outside of the 95% CI, but especially for the PMF on extreme contrasts this rate is elevated. Also note that large differences are not necessarily problematic, as behaviour can simply be noisy.

Full list of International Brain Laboratory members

Larry Abbot, Luigi Acerbi, Valeria Aguillon-Rodriguez , Mandana Ahmadi, Jaweria Amjad, Dora Angelaki, Jaime Arlandis, Zoe C. Ashwood, Kush Banga, Hailey Barrell, Hannah M. Bayer, Brandon Benson, Julius Benson, Jai Bhagat, Dan Birman, Niccolò Bonacchi, Kcenia Bougrova, Julien Boussard, Sebastian A. Bruijns, E Kelly. Buchanan, Robert Campbell, Matteo Carandini, Joana A. Catarino, Fanny Cazettes, Gaelle A. Chapuis, Anne K. Churchland, Davide Crombie, Yang Dan, Felicia Davatolhagh, Peter Dayan, Eric EJ. DeWitt, Sophie Denève, Tatiana Engel, Michele Fabbri, Mayo Faulkner, Robert Fetcho, Ila Fiete, Charles Findling, Laura Freitas-Silva, Surya Ganguli, Berk Gercek, Naureen Ghani, Ivan Gordeliy, Laura M. Haetzel, Kenneth D. Harris , Michael Hausser, Naoki Hiratani, Sonja Hofer, Fei Hu, Felix Huber, Julia M. Huntenburg, Cole Hurwitz, Anup Khanal, Christopher S. Krasniak, Christopher S. Krasniak, Sanjukta Krishnagopal, Michael Krumin, Debottam Kundu, Agnès Landemard, Christopher Langdon, Christopher Langfield, Inês C. Laranjeira, Peter Latham, Petrina Lau, Hyun Dong. Lee, Ari Liu, Zachary F. Mainen, Amalia Makri-Cottington, Hernando Martinez-Vergara, Brenna McMannon, Isaiah McRoberts, Guido T. Meijer, Maxwell Melin, Leenoy Meshulam, Kim Miller, Nathaniel J. Miska, Catalin Mitelut, Zeinab Mohammadi, Thomas Mrsic-Flogel, Masayoshi Murakami, Jean-Paul Noel, Kai Nylund, Farideh Oloomi, Liam Paninski, Sabrina Perrenoud, Alberto Pezzotta, Samuel Picard, Jonathan W. Pillow, Alexandre Pouget, Carolina Quadrado, Pranav Rai, Georg Raiser, Florian Rau, Cyrille Rossant, Noam Roth, Nicholas A. Roy, Kamron Saniee, Rylan Schaeffer, Michael M. Schartner, Yanliang Shi, Karolina Z. Socha, Cristian Soitu, Nicholas A. Steinmetz, Karel Svoboda, Marsa Taheri, Charline Tessereau, Matthew Tucker, Anne E. Urai, Erdem Varol, Alejandro Pan Vazquez, Shuqi Wang, Miles J. Wells, Steven J. West, Matthew R. Whiteway, Charles Windolf, Olivier Winter, Ilana Witten, Lauren E. Wool, Zekai Xu, Kenneth Yang, Yaxuan Yang, Han Yu, Anthony M. Zador, Yizi Zhang
