# Supplementary material for: Infinite hidden Markov models can dissect the complexities of learning
Source: Nat Neurosci. 2025 Dec 30;29(1):186–94. doi: 10.1038/s41593-025-02130-x (PMC12779568; doi:10.1038/s41593-025-02130-x)
Supplement: Supplementary file 2 — Reporting Summary [file 41593_2025_2130_MOESM2_ESM.pdf]

nature portfolio

Corresponding author(s): Sebastian Bruijns

Last updated by author(s): Oct 2, 2025

Reporting Summary

Nature Portfolio wishes to improve the reproducibility of the work that we publish. This form provides structure for consistency and transparency in reporting. For further information on Nature Portfolio policies, see our Editorial Policies and the Editorial Policy Checklist.

Statistics

For all statistical analyses, confirm that the following items are present in the figure legend, table legend, main text, or Methods section.

n/a	Confirmed	
☐	☒	The exact sample size (*n*) for each experimental group/condition, given as a discrete number and unit of measurement
☐	☒	A statement on whether measurements were taken from distinct samples or whether the same sample was measured repeatedly
☐	☒	The statistical test(s) used AND whether they are one- or two-sided<br>*Only common tests should be described solely by name; describe more complex techniques in the Methods section.*
☐	☒	A description of all covariates tested
☐	☒	A description of any assumptions or corrections, such as tests of normality and adjustment for multiple comparisons
☐	☒	A full description of the statistical parameters including central tendency (e.g. means) or other basic estimates (e.g. regression coefficient) AND variation (e.g. standard deviation) or associated estimates of uncertainty (e.g. confidence intervals)
☐	☒	For null hypothesis testing, the test statistic (e.g. *F*, *t*, *r*) with confidence intervals, effect sizes, degrees of freedom and *P* value noted<br>*Give P values as exact values whenever suitable.*
☐	☒	For Bayesian analysis, information on the choice of priors and Markov chain Monte Carlo settings
☐	☒	For hierarchical and complex designs, identification of the appropriate level for tests and full reporting of outcomes
☐	☒	Estimates of effect sizes (e.g. Cohen's *d*, Pearson's *r*), indicating how they were calculated

Our web collection on statistics for biologists contains articles on many of the points above.

Software and code

Policy information about availability of computer code

Data collection

https://github.com/int-brain-lab/iblrig contains the code running on data collection rigs, precise task protocol identifiers are listed within the dataset.

Data analysis

Our analysis code can be found at the repository linked below. We make use of Markov chain Monte-Carlo algorithms, from these packages:

pybasicbayes (original version='0.2.4', our version linked below)
pyhsmm (original version='0.1.6', our version linked below)
pypolyagamma (version='1.2.3')

Code availability:

The analysis code with installation instructions is deposited at https://github.com/SebastianBruijns/diHMM

This uses:

https://github.com/SebastianBruijns/sab_pybasicbayes a modified version of https://github.com/mattjj/pybasicbayes

https://github.com/SebastianBruijns/sab_pyhsmm a modified version of https://github.com/mattjj/pyhsmm

For manuscripts utilizing custom algorithms or software that are central to the research but not yet described in published literature, software must be made available to editors and reviewers. We strongly encourage code deposition in a community repository (e.g. GitHub). See the Nature Portfolio guidelines for submitting code & software for further information.

Data

Policy information about availability of data

All manuscripts must include a data availability statement. This statement should provide the following information, where applicable:

- Accession codes, unique identifiers, or web links for publicly available datasets
- A description of any restrictions on data availability
- For clinical datasets or third party data, please ensure that the statement adheres to our policy

Please follow these: https://int-brain-lab.github.io/iblenv/notebooks_external/data_download.html instructions to download the data used in this article. Use for example the following code snippet to download the data using Python.

```
from one.api import ONE
import re

# use password as indicated on the website
one = ONE(base_url='https://openalyx.internationalbrainlab.org', password='*****')

regexp = re.compile(r'Subjects/\w*/((\w|-)+)/_ibl')
datasets = one.alyx.rest('datasets', 'list', tag='2023_Q4_Bruijns_et_al')

# extract subject names
subjects = [regexp.search(ds['file_records'][0]['relative_path']).group(1) for ds in datasets]
# reduce to list of unique names
subjects = list(set(subjects))

for subject in subjects:
    trials = one.load_aggregate('subjects', subject, '_ibl_subjectTrials.table')
    training = one.load_aggregate('subjects', subject, '_ibl_subjectTraining.table')
    # save data
```

Research involving human participants, their data, or biological material

Policy information about studies with human participants or human data. See also policy information about sex, gender (identity/presentation), and sexual orientation and race, ethnicity and racism.

Reporting on sex and gender	N/A
Reporting on race, ethnicity, or other socially relevant groupings	N/A
Population characteristics	N/A
Recruitment	N/A
Ethics oversight	N/A

Note that full information on the approval of the study protocol must also be provided in the manuscript.

Field-specific reporting

Please select the one below that is the best fit for your research. If you are not sure, read the appropriate sections before making your selection.

☐ Life sciences ☒ Behavioural & social sciences ☐ Ecological, evolutionary & environmental sciences

For a reference copy of the document with all sections, see nature.com/documents/nr-reporting-summary-flat.pdf

Behavioural & social sciences study design

All studies must disclose on these points even when the disclosure is negative.

Study description	Mice were taught a perceptual decision-making task: On each trial, a patch of black bars was presented on a white background, on either the right or left side of a screen. Mice used a wheel to indicate which side the contrast was on, for a water reward if correct. By modulating the strength of the contrast, a trial could be made more or less difficult. Mice were only presented easy contrasts at the start, more difficult contrasts were introduced as performance improved. This gave us a quantitative experimental study.
Research sample	We analysed 134 C57BL6/J mice aged 3-7 months obtained from Jackson Laboratory or Charles River. We used the publicly available

Research sample	IBL dataset and included all subjects. We did not therefore determine the data collection ourselves, but relied on an existing, exceptionally large data set. In particular, the number of individuals is larger than that used by the studies of e.g. Kastner et al. (2022) or Akiti et al. (2022).
Sampling strategy	Our sampling strategy was convenience/exhaustive. To our knowledge we used all mice which trained under the standard IBL protocol without any manipulations, but we did not make entirely sure that none were missed. We did not specifically leave out any appropriate mice, but we did exclude mice which had incomplete training trajectories (missing sessions for whatever reason).
Data collection	Data was collected using the IBL rig (https://github.com/int-brain-lab/iblrig) setup, in particular mouse responses were recorded via computer. All the details can be found in the paper describing the experiment setup: https://elifesciences.org/articles/63711. Researchers were not blind to experimental condition, as there were no conditions. Researchers were effectively blind to the study hypothesis, as hypotheses were formed during model construction, which was mostly after data collection had concluded.
Timing	Samples were collected beginning on the 3rd of November 2019 and ending on the 8th of April 2022.
Data exclusions	12 subjects were excluded from the analysis because the R^hat metric was too bad (above 1.05) on their chains, as described in the paper. The R^hat metric quantifies how much the chains vary from one another, and indicate poor convergence. We also excluded mice with any missing training sessions.
Non-participation	We analysed mice which completed training, in that sense there were no dropouts.
Randomization	There were no experimental groups.

Reporting for specific materials, systems and methods

We require information from authors about some types of materials, experimental systems and methods used in many studies. Here, indicate whether each material, system or method listed is relevant to your study. If you are not sure if a list item applies to your research, read the appropriate section before selecting a response.

Materials & experimental systems

n/a	Involved in the study	
☒	☐	Antibodies
☒	☐	Eukaryotic cell lines
☒	☐	Palaeontology and archaeology
☐	☒	Animals and other organisms
☒	☐	Clinical data
☒	☐	Dual use research of concern
☒	☐	Plants

Methods

n/a	Involved in the study	
☒	☐	ChIP-seq
☒	☐	Flow cytometry
☒	☐	MRI-based neuroimaging

Animals and other research organisms

Policy information about studies involving animals; ARRIVE guidelines recommended for reporting animal research, and Sex and Gender in Research

Laboratory animals	C57BL6/J mice aged 3-7 months obtained from Jackson Laboratory or Charles River.
Wild animals	Study did not involve wild animals.
Reporting on sex	Sex was not considered in this study. We wanted to consider learning in general.
Field-collected samples	Study did not involve samples collected from the field.
Ethics oversight	All procedures and experiments were carried out in accordance with the local laws and following approval by the relevant institutions: the Animal Welfare Ethical Review Body of University College London [P1DB285D8]; the Institutional Animal Care and Use Committees of Cold Spring Harbor Laboratory [1411117; 19.5], Princeton University [1876-20], and University of California at Berkeley [AUP-2016-06-8860-1]; the University Animal Welfare Committee of New York University [18-1502]; and the Portuguese Veterinary General Board [0421/0000/0000/2016-2019].

Note that full information on the approval of the study protocol must also be provided in the manuscript.

Plants

Seed stocks

Report on the source of all seed stocks or other plant material used. If applicable, state the seed stock centre and catalogue number. If plant specimens were collected from the field, describe the collection location, date and sampling procedures.

Novel plant genotypes

Describe the methods by which all novel plant genotypes were produced. This includes those generated by transgenic approaches, gene editing, chemical/radiation-based mutagenesis and hybridization. For transgenic lines, describe the transformation method, the number of independent lines analyzed and the generation upon which experiments were performed. For gene-edited lines, describe the editor used, the endogenous sequence targeted for editing, the targeting guide RNA sequence (if applicable) and how the editor was applied.

Authentication

Describe any authentication procedures for each seed stock used or novel genotype generated. Describe any experiments used to assess the effect of a mutation and, where applicable, how potential secondary effects (e.g. second site T-DNA insertions, mosiacism, off-target gene editing) were examined.
